# Introgressed Saltol QTL Lines Improves the Salinity Tolerance in Rice at Seedling Stage

**DOI:** 10.3389/fpls.2020.00833

**Published:** 2020-06-11

**Authors:** S. L. Krishnamurthy, Preeti Pundir, Arvinder Singh Warraich, Suman Rathor, B. M. Lokeshkumar, Nagendra Kumar Singh, Parbodh Chander Sharma

**Affiliations:** ^1^Division of Crop Improvement, ICAR-Central Soil Salinity Research Institute, Karnal, India; ^2^Rice Genomics Laboratory, ICAR-National Institute for Plant Biotechnology, New Delhi, India

**Keywords:** salinity, *sice*, *Saltol* QTL, NILs, seedling stage, marker assisted backcross breeding

## Abstract

Rice is a staple food crop in Asia and plays a crucial role in the economy of this region. However, production of rice and its cultivating areas are under constant threat of soil salinity. A major QTL, *Saltol*, responsible for salinity tolerance at seedling stage has been mapped on chromosome 1 using Pokkali/IR29 Recombinant Inbred Lines (RIL) population. The present study was aimed to incorporate *Saltol* Quantitative Trait Loci (QTL) in two high yielding mega rice varieties i.e. Pusa44 and Sarjoo52 through Marker Assisted Backcross Breeding (MABB). To improve the seedling stage salinity tolerance in these cultivars, we introgressed the *Saltol* QTL from donor parent FL478 a derivative of Pokkali. A total of three backcrosses (BC_3_) followed by selfing have led to successful introgression of *Saltol* QTL. Foreground selection at each breeding cycle was done using micro-satellite markers RM3412 and AP3206 to confirm *Saltol* QTL. The precise transfer of *Saltol* region was established using recombinant selection through flanking markers RM493 and G11a. Finally, 10 *Saltol* near isogenic lines (NILs) of Pusa44 and eight NILs of Sarjoo52 were successfully developed. These NILs (BC_3_F_4_) were evaluated for seedling stage salinity under hydroponic system. The NILs PU99, PU176, PU200, PU215, PU229, PU240, PU241, PU244, PU252, PU263 of Pusa44 and SAR17, SAR23, SAR35, SAR39, SAR77, SAR87, SAR123, SAR136 NILs of Sarjoo52 confirmed tolerance to salinity with low salt injury score of 3 or 5. Ratio of Na^+^/K^+^ content of *Saltol* NILs ranged from 1.26 to 1.85 in Pusa44 and 1.08 to 1.69 in Sarjoo52. The successfully developed NILs were further phenotyped stringently for morphological traits to estimate Phenotypic Recovery. Background selection of NILs along with parents was carried out with 50K SNP chip and recovered 94.83–98.38% in Pusa44 NILs and 94.51 to 98.31% in Sarjoo52 NILs of recurrent genome. The present study of MAB has accelerated the development of salt tolerant lines in the genetic background of Pusa44 and Sarjoo52. These NILs could be used for commercial cultivation in saline affected area.

## Introduction

Rice is an important cereal crop that is widely produced for human consumption. It is staple food crop for half of the world population and in India it is a key component of food security programs. Moreover, India is largest exporter of rice and exported 12.5 million metric tons (Shahbandeh, 2019) which contributes significantly to the Indian economy. However, abiotic stresses critically limit the crop production and causes significant yield losses. Soil salinity is second major abiotic threat to rice production after drought. Soil salinity is expected to increase globally due to climate change and conventional irrigation practices. More than 800 million hectares of world's land area is salt affected which constitutes more than 6% of world land area (FAO, 2014). In India salt affected land accounts for 6.73 million hectares which is expected to increase to 16.2 million hectares by 2050 (CSSRI Vision-2050, 2015; Singh, 2018). Salinity could also be attributed to rising sea level which is bringing saline water to inlands and exposing rice growing areas to saline conditions. Due to salinity most of the rice suited areas are left uncultivated or rice grown under these areas has very less yield/hectare compared to national average yield. Rice is considered salt sensitive crop and it is mostly affected at seedling and reproductive stage of its life cycle (Mass and Hoffman, 1977; Zeng et al., 2001; Singh et al., 2007; Tiwari et al., 2015; Krishnamurthy et al., 2016a). Salinity exposure leads to immediate effect which is manifested in few days to some long term affects which occurs after several days to week from initial exposure to salt (Roy et al., 2014; Krishnamurthy et al., 2016c). Upon initial exposure to salinity, plants stomata closes due to change in water potential which leads to inhibition of shoot elongation. This phenomenon is salt accumulation independent effect and was termed osmotic phase in rice plant by Munns and Tester (2008). Long term exposure to salinity was termed ionic phase which is characterized by Na^+^ accumulation and premature senescence of older leaves (Singh et al., 2014; Reddy et al., 2017).

The salinity tolerance plays a crucial role in rice productivity (Momayezi et al., 2009; Tack et al., 2015). Salinity tolerance in rice is a multifactorial trait highly influenced by environment. Genotype and environmental interaction play an important role for salinity tolerance (Krishnamurthy et al., 2016b; Krishnamurthy et al., 2016d; Krishnamurthy et al., 2017). At seedling stage, salinity leads to poor establishment of rice, diminished root/shoot length, leaf size reduction which leads to early plant death (Zeng and Shannon, 2000; Krishnamurthy et al., 2016c). At reproductive stage, salinity tends to reduce the yield by affecting several yield contributing factors (Khatun et al., 1995; Grattan et al., 2002; Singh et al., 2004; Kumar et al., 2015; Krishnamurthy et al., 2015b). Therefore seedling stage salinity tolerance is very important for early plant establishment under saline stress which could help the plant to achieve good vegetative growth later (Babu et al., 2017a). Rice plant has several mechanisms to tolerate high salt concentration; these mechanisms are ion exclusion, osmotic tolerance and tissue tolerance (Munns and Tester, 2008; Roy et al., 2014). Ion exclusion works at root level to prevent the excess accumulation of Na^+^ and Cl^−^ in leaves (Yeo et al., 1990). Osmotic tolerance involves the ability of rice plant to tolerate the drought aspect of salinity by maintaining leaf expansion and stomatal conductance (Rajendran et al., 2009). Tissue tolerance involves sequestration of Na^+^ in the vacuole, enzymes production that catalyze detoxification of reactive oxygen species (ROS) and synthesis of compatible solutes that helps in maintaining positive water potential. There are some traditional landraces like Pokkali, Nona Bokra and Kalaratta in their natural saline habitat exhibits one of these mechanisms and are highly adaptable to these conditions. However, regardless of their ability to survive salinity, these landraces cannot be adopted in commercial production due to their poor quality of seed and less yield. But these genotypes have proved to be an excellent source of salt tolerance gene that could be used to develop salt tolerant varieties (Ravikiran et al., 2018). An important seedling stage salinity tolerant variety FL478 was developed using a high salt tolerant landrace Pokkali. Later, the RILs developed from the parents (Pokkali and IR29) helped in discovery of major QTL *Saltol* on chromosome 1 (Bonilla et al., 2002). This QTL explained phenotypic variation of 43% for seedling stage shoot Na^+/^K^+^ homoeostasis and express the tolerance at seedling stage in rice. Salinity tolerance at seedling stage is important to establishment of crop at the early stage which is pre requisite for higher grain yield. The salinity tolerance at seedling stage is important in rice where cultivating ecology is coastal area. In coastal salinity is one of the main important stresses at seedling stage. Salinity tolerant at seedling stage is also important where practicing of Direct Seeded Rice (DSR) at salt affected areas.

To counter the damaging effects of salinity on rice production new improved salt tolerant varieties are being developed through conventional breeding method (Krishnamurthy et al., 2019a; Krishnamurthy et al., 2019b; Krishnamurthy et al., 2019c) and also through marker assisted breeding (Singh et al., 2016; Babu et al., 2017b; Geetha et al., 2017; Singh et al., 2018; Bhandari et al., 2019). The conventional method of breeding however is time consuming and more labour intensive (Salvi and Tuberosa, 2005). But MAB on the other hand is more precise and faster method for introgression of useful genes. MAB allows selection at each breeding cycle to validate precise transfer of gene and it also allows limiting the donor region, therefore, avoiding any linkage drag (Singh et al 2016). Finally, the recovery of developed lines can be calculated to mark the high recurrent parental genome recovery (RPG). The DUS (distinctness, uniformity, and stability) testing of NILs along with parents helps to know the recovery of recurrent parental traits along with 50K SNP genotyping (Mani et al., 2015; Singh et al., 2015). Haplotype of *Saltol* QTL in rice helps to identify the novel salt tolerant genotypes and are helpful in molecular breeding programs to enhance the salinity tolerance in rice (Ali et al., 2013; Babu et al., 2014; Krishnamurthy et al., 2014; Krishnamurthy et al., 2015a; Choudary et al., 2016; Ravikiran et al., 2018).

Marker assisted breeding in rice was successful in developing new improved salt tolerance line in rice (Linh et al., 2012; Luu et al., 2012; Usatov et al., 2015; Singh et al., 2016; Babu et al., 2017b; Singh et al., 2018; Bhandari et al., 2019). Molecular markers based technologies helped in mapping of salt tolerant genes on rice chromosomes and few *Saltol* linked marker like RM3412, AP3206, RM8094, RM493, RM10793 have been identified for marker assisted breeding and screening (Ismail et al., 2007; Thomson et al., 2010). Rice mega varieties namely, Pusa44 and Sarjoo52 are the most popular varieties in the North Western region of India where the green revolution was initiated in rice. These mega rice varieties are high yield varieties under non saline stress situation whereas, highly sensitive to salinity stress at seedling stage. Therefore, the markers assisted breeding (MAB) breeding was employed for introgression of Saltol QTL into these rice varieties to enhance their salinity tolerance.

## Materials and Methods

### Plant Material

**Figure 1:**
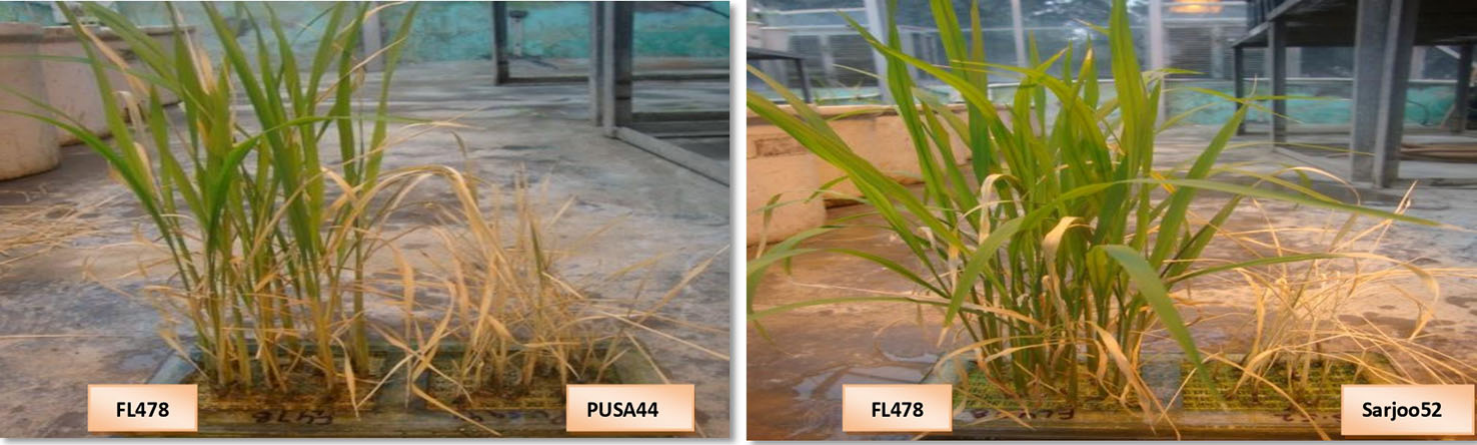
Seedling stage salinity screening of FL478, Sarjoo52 and Pusa44.

In the present investigation two high yielding salt sensitive mega varieties of rice Pusa44 and Sarjoo 52 used as recurrent parents to introgress *Saltol* QTL,from FL478, a seedling stage salinity tolerant variety was used as donor parent. Both Pusa44 and Sarjoo52 cultivars were selected as recipient parents because of their good agronomic characters and suitability in rice growing areas (Mandal et al., 2018). Parents were evaluated for salt tolerance at 8.0 dSm^−1^ in hydroponics and it was found that both Pusa44 and Sarjoo52 were highly susceptible to salinity, FL478 withstands salt very efficiently with very less visual salt injury symptoms (**Figure 1**). The crosses between recurrent and donor parents were made at Indian Council of Agriculture Research-Central Soil Salinity Research Institute (ICAR-CSSRI) (29° 42' 31.13'') (76° 57' 2.13''), Karnal and advanced during *Kharif (*June–September*)* season in field and summer (March to May) off-season in glass house conditions from 2010 to 2018.

### Molecular Analysis

Genomic DNA was isolated from rice seedlings using modified Cetyl Trimethyl Ammonium Bromide (CTAB) method (Zheng et al., 1995). The DNA concentration was diluted to 30 ng μl^−1^ by UV–vis spectrophotometer (NanoDrop 2000c, Thermo Scientific Products, Wilmington, USA). PCR reactions were carried out on Biometra TGradient Thermocycler (Imperial Life Science (P) Limited, Gurgaon, India). Polymerase chain reaction (PCR) based amplification of target genomic region was done using selected markers in a 10 μl reaction mixture containing 30–50 ng of genomic DNA, 10 pmol each of both forward and reverse primers (Sigma Aldrich, Bangalore, India), 0.2 mM dNTPs, 1.5 mM of MgCl_2_ and 1.0 U of Taq polymerase (Merck Specialities Private Limited, Mumbai, India). The PCR was run for 35 cycles with initial denaturation for 5 min at 95 °C, followed by 35 cycles of 30 s of denaturation at 95 °C, 30s annealing at 55 °C and extension for 1 min at 72 °C. After completion of 35 cycles, final extension at 72 °C for 7 min and kept at 4 °C for cool down. The amplified product was mixed with tracking dye and resolved in gel electrophoresis (CBS Scientific, Thermo Scientific Products, Wilmington, USA) on 3% agarose gel (MP Biomedicals, LLC, Mumbai, India) in 1 × TBE buffer along with DNA ladder (Thermo Scientific, Wilmington, USA). The gel was scanned in gel documentation system (FluorChem HD2 system, USA) and polymorphic bands were scored for further analysis. Foreground microsatellite markers RM3412 and AP3206 which tightly linked to *Saltol* QTL (Thompson et al., 2010; Babu et al., 2014), while recombinant markers RM493 and G11A flanked to *Saltol* QTL (Linh et al., 2012) are employed during foreground and recombinant selection programme. 50K SNP genotyping (Singh et al., 2015) was used to confirm the retaining of maximum desirable genome of recurrent parents in selected salt tolerant NILs.

### Breeding Strategy

The parents were initially screened for three *Saltol* markers namely, RM3412, AP3206 and RM10793 (Thomson et al., 2010; Babu et al., 2016). Out of which two markers (RM3412 and AP3206) were found to be polymorphic between recurrent and donor parents. These microsatellite markers (RM3412 and AP3206) were used for foreground selection, while flanking markers RM493 and G11A were used for recombinant selection to minimize the donor segment (**Table 1**). The present study was carried out from 2010 to 2018, donor parent FL478 was crossed with Pusa44 and Sarjoo52 during *Kharif (June–September) 2010*. The hybridity of F_1_s was confirmed using *Saltol*-linked SSR marker RM3412 and AP3206 as part of foreground selection. The confirmed F_1_s were back crossed to their respective recurrent parent to generate BC_1_F_1_ seeds. The BC_1_F_1_ population was subjected to screening for heterozygous *Saltol* region and recombinant selection; the selected plants from BC_1_F_1_ populations were advanced to BC_2_F_1_. The breeding cycle was repeated until BC_3_F_1_ with careful marker assisted foreground and recombination selection of progenie. At BC_3_F_1_ plants exhibiting homozygosity for recombinant marker RM493 and G11A were selected from both the populations (Pusa44 and Sarjoo52) and advanced to BC_3_F_2_ by selfing. In the BC_3_F_2_ generation, the individual plants showing homozygygosity for *Saltol* QTL were selected and finally advanced to BC_3_F_4_.

**Table 1:** List of Saltol SSR markers, forward and reverse sequences used for foreground and recombinant selection in development of *Saltol* NILs of Pusa44 and Sajoo502.

Sr. No.	Markers	Position (mb)	Forward primer	Reverse primer	Remarks
1	G11A	10.24	AGCTGGTAGGAAGGCTGAAAG	TGCCAGGCAGCTCAGTAGAAG	Recombinant marker
2	AP3206	11.23	TTCTCATCGCACCATCTCTG	CGACGAGGAGAGGAAGAAG	Foreground marker
3	RM3412	11.58	AAAGCAGGTTTTCCTCCTCC	CCCATGTGCAATGTGTCTTC	Foreground marker
4	RM493	12.28	GTACGTAAACGCGGAAGGTGACG	CGACGTACGAGATGCCGATCC	Recombinant marker

### Screening for Seedling Stage Salinity Tolerance

The developed NILs of Pusa44 and Sarjoo52 which were homozygous for *Saltol* QTLs were screened using hydroponics for seedling stage salinity tolerance along with respective parents under controlled glass house conditions at ICAR-CSSRI, Karnal, India, with 29-35°C day/ 21°C night temperature. Relative humidity was kept at 30–40% and photoperiod of 13 h. The 200 L nutrient tanks were filled with Yoshida nutrient solution (Yoshida et al., 1976) and seedlings were established on floating grids. The whole screening material was divided in to two sets using three replications in each set, one set for control (non-stress) environment and other set for saline treatment. The seeds were sown in hydroponics on the floating grid using normal water for first three days to germinate, after germination plants along with floating grids were transferred to nutrient medium. The solution was replaced once in a week and pH was maintained at 4.5–5.0 (IRRI, 2013). After the 14th day of sowing, imposed the saline stress through addition of measured quantity of salt (Sodium Chloride (NaCl)) to nutrient solution and maintained salinity stress level of EC ~8.0 dSm^−1^. Under microplot screening, the seeds of NILs along with parents were allowed to germinate in the soils. Saline water (EC ~8.0 dSm^−1^) was irrigated to the soils in microplot for desired salinity stress level (EC ~8.0 dSm^−1^). After stringent phenotypic screening of developed NILs under salt stress conditions, the NILs from both populations exhibited tolerance and moderate tolerance were selected and genotyped for background selection using 50K SNP chip. The 50K SNP chip included 50,051 SNPs from 18,980 different genes covering all 12 chromosomes in rice, including 3,710 single-copy (SC) genes conserved between wheat and rice, 14,959 SC genes unique to rice, 194 agronomically important cloned rice genes and 117 multi-copy rice genes. The 50K SNP chip used in rice Germplasm characterisation, association mapping, background selection and evolutionary studies as it is efficient and reliable tool (Singh et al., 2015). SNP genotyping helped to confirm the maximum background recovery of desirable recurrent parental genome in the selected NILs at molecular level.

### Measurement of Morphological and Physiological Traits

Phenotyping of NILs along with parents was carried out in randomized complete block design (RCBD) three replications in hydroponics and micro plot during Kharif (*June–September*) 2015 and 2016, respectively. To evaluate the salinity tolerance of *Saltol* NILs (Pusa44 and Sarjoo52) 5 traits were recorded namely salt injury score, root length (cm), shoot length (cm), Na^+^ and K^+^ concentration (mM g dw^−1^) and Na^+^/K^+^. Shoot length was measured from the base of the plant to the tip of longest leaf and root length was measured from the base of the plant to the tip of longest root. For seedling score, the seedlings after 14 days exposure to salt stress were scored as per standard evaluation system (SES) score for rice (IRRI, 2013). The salt injury score 1 was treated as highly tolerant, 3 was tolerant, 5 was moderately tolerant, 7 was susceptible and 9 was highly susceptible. The concentration of Na^+^ and K^+^ ions of plant samples were estimated from di-acid digestion (HNO_3_:HClO_4_ 3:1) of samples using atomic absorption spectroscopy (AAS-Zeenit 700P, Analytik Jena, Germany) as per the protocol of Miller and Rutzke 2003.

### Distinctive, Uniformity and Stability (DUS)-Based Characterization of NILs

The NILs of Pusa44 and Sarjoo52 were evaluated for distinctness, uniformity and stability (DUS) characters along with recurrent parents to find the percent similarity (Mondal et al., 2014; Mani et al., 2015). DUS testing is a mode of determining whether a newly bred NILs differs from recurrent parents within the same species (the Distinctness), whether the traits used to establish Distinctness are expressed uniformly (the Uniformity) and that these traits do not change over subsequent generations (the Stability). The *Saltol* introgressed NILs along with their parents were transplanted from nursery to field at spacing 20 cm × 15 cm in augmented design experiment during *Kharif* (*June–September*) season of 2016, 2017 and 2018 at the research farm of ICAR-CSSRI, Karnal. The crops were raised as per standard agronomic package of practice and data were recorded at the different stages and characters of NILs were compared with that of recurrent parent (**Supplementary Table 1**). Similarity percentage of NILs towards the recurrent parent was calculated.

## Results

### Development of NILs by Marker-Assisted Back Cross Selection

**Figure 2:**
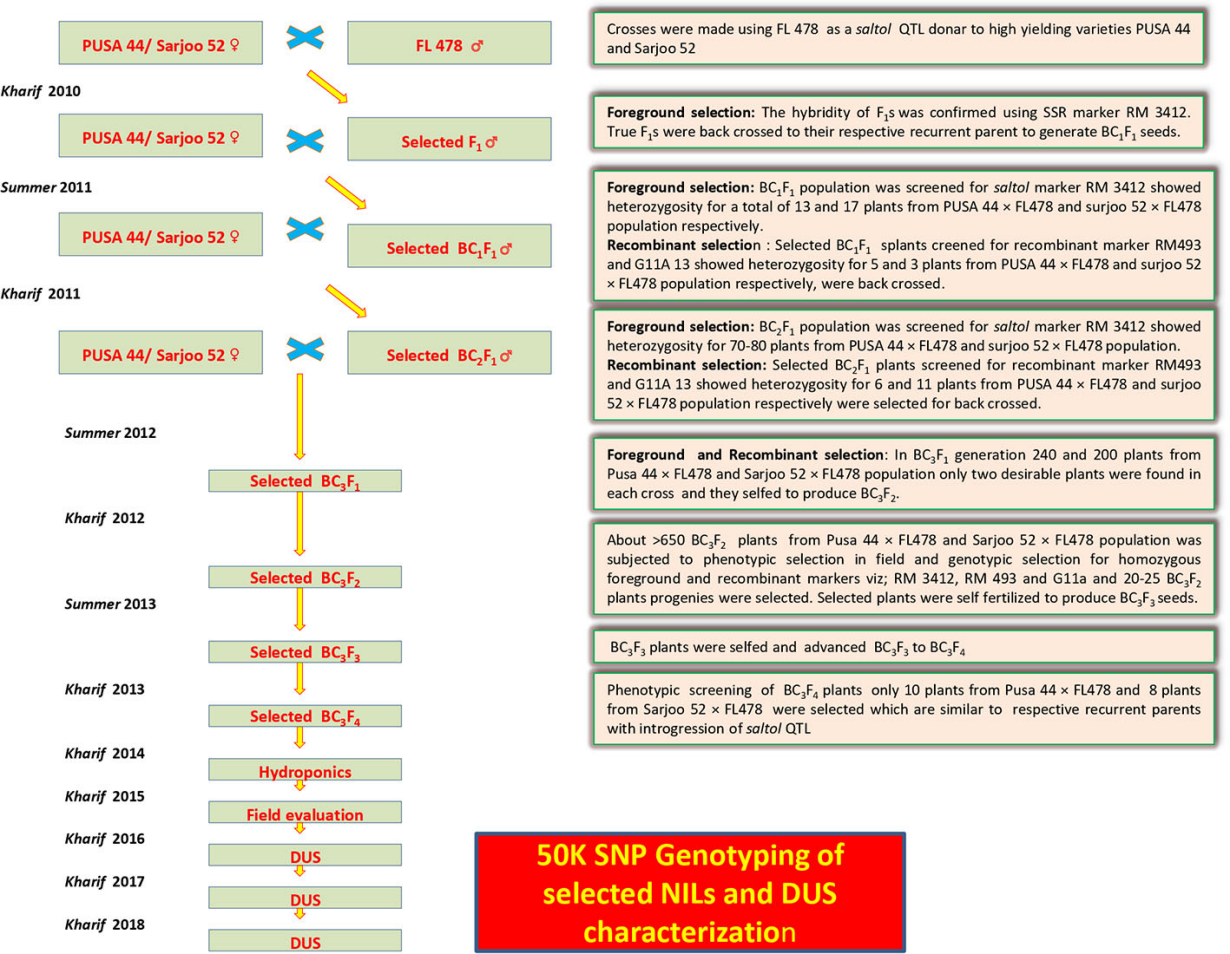
Flowchart for breeding scheme used in marker assisted breeding for successful introgresion of *Saltol* locus from donor parent to elite breeding lines of Pusa44 and Sarjoo52.

**Figure 3:**
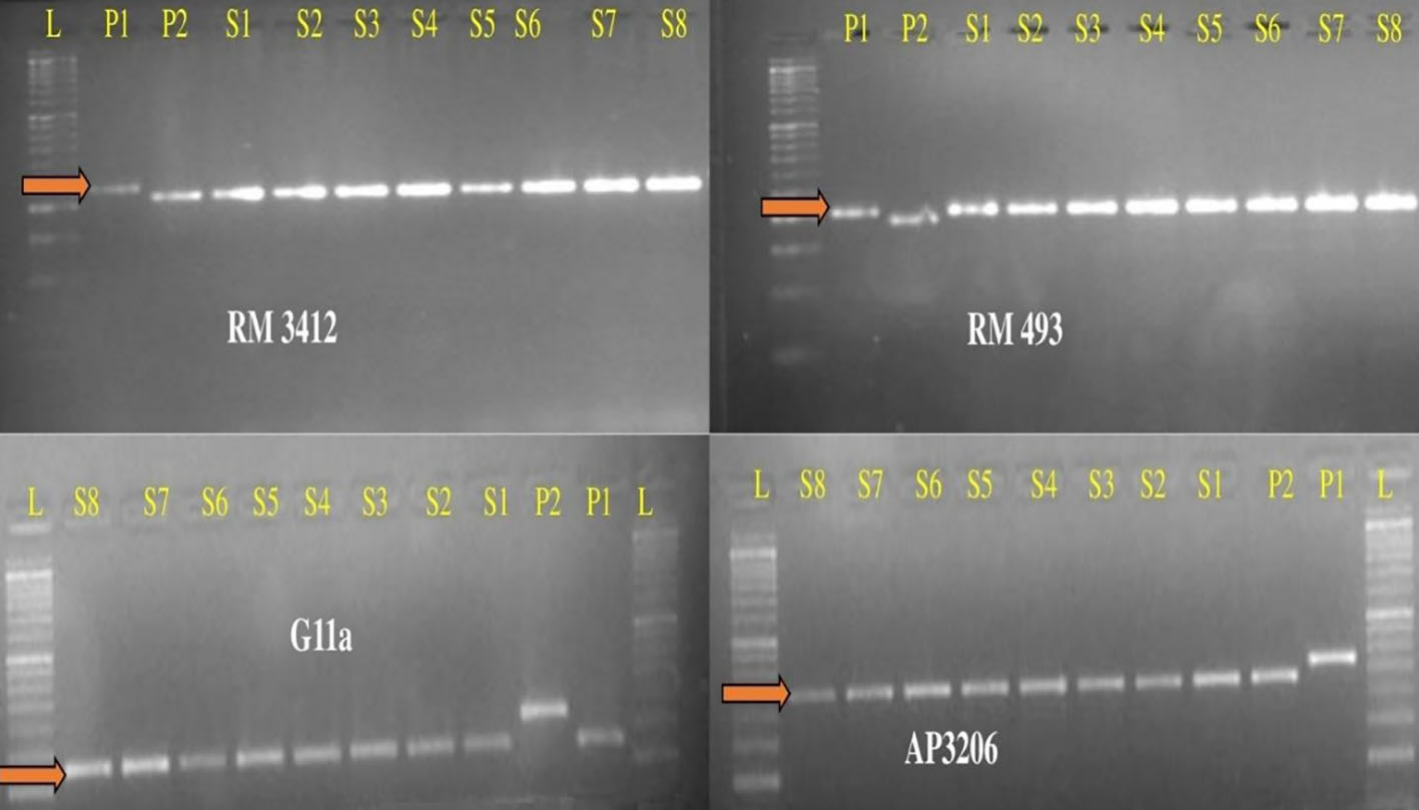
Gel images of BC_3_F_4_ NILs of Pusa44 based on foreground and recombinant marker screening. L-50bp Ladder, K1-Pusa44, K2-FL478, P1-PU99, P2-PU176, P3-PU200, P4-PU215, P5-PU229, P6-PU240, P7-PU241, P8-PU244, P9-PU252 and P10-PU263.

**Figure 4:**
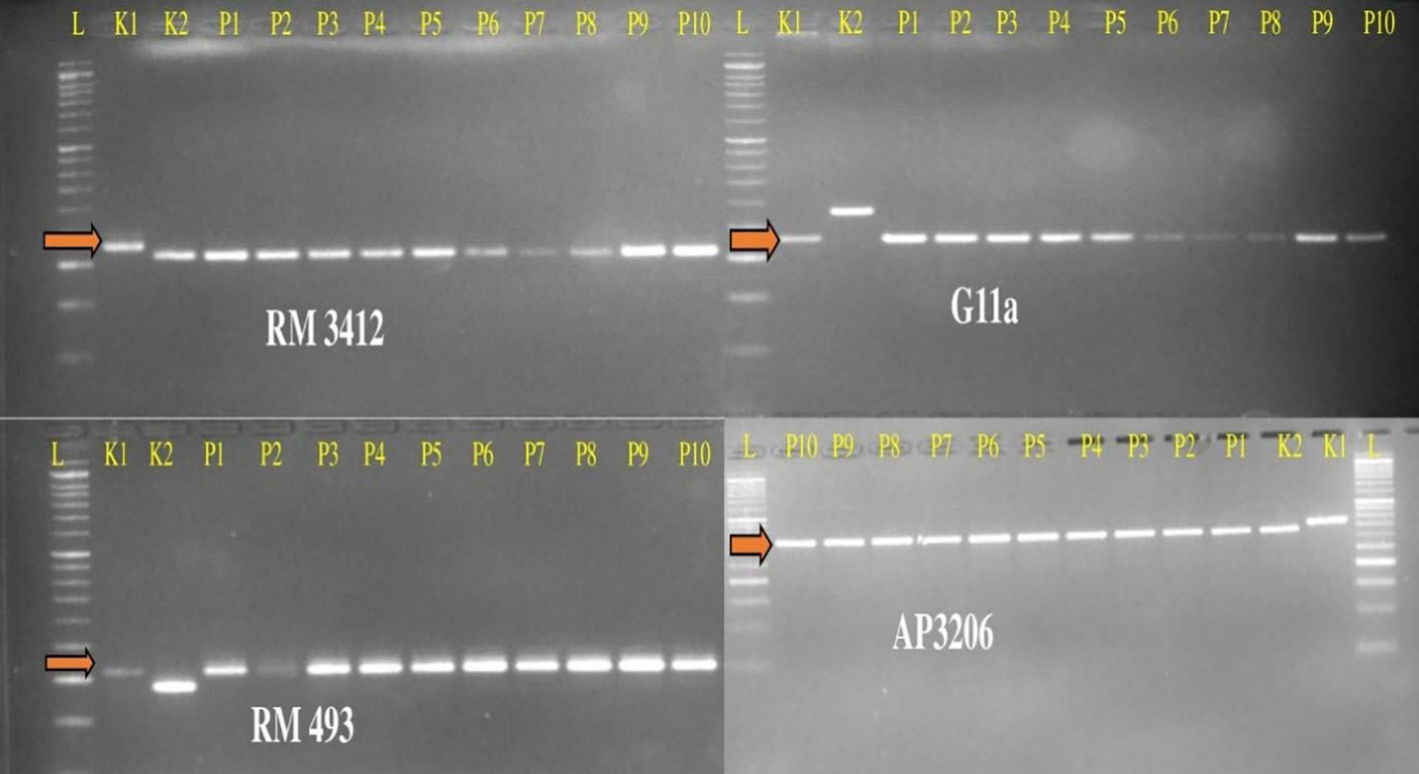
Gel images of BC_3_F_4_ NILs of Sarjoo52 based on foreground and recombinant marker screening. L-50bp Ladder, P1-Sarjoo52, P2-FL478, S1-SAR17, S2-SAR23, S3-SAR35, S4-SAR56, S5-SAR77, S6-SAR87, S7-SAR123, and S8-SAR136.

The present study leads to the development of *Saltol* NILs of Pusa44 and Sarjoo52 through Marker Assisted Backcross Breeding (MABB) scheme (**Figure 2**). Out of *Saltol* linked markers, there were four markers which were polymorphic between the parents (FL478, Pusa44 and Sarjoo52) two of them used for foreground selection and other two used for recombination selection. Crosses were made during the cropping season using staggered sowing to match the flowering time and 250 F_1_ seeds were harvested from cross Pusa44 × FL478 and 150 F_1_ seeds were harvested from Sarjoo52 × FL478 cross. The true hybridity of F_1_ is confirmed by employing *Saltol* linked markers RM3412 and AP3206. The true F_1_s were selected from both the population and subjected to backcrossing with respective recurrent parents. A total of 407 BC_1_F_1_ seeds were harvested from Pusa44 × F_1_ cross and 938 BC_1_F_1_ seeds were harvested from Sarjoo52 × F_1_ cross. Foreground screening of BC_1_F_1_ population with markers RM3412 and AP3206 showed heterozygosity in 13 BC_1_F_1_ plants of Pusa44 back cross progenies and 17 BC_1_F_1_ plants in Sarjoo52 back cross progenies. The recombinant selection was employed using recombinant markers RM493 and G11A to limit the donor region beyond *Saltol* region, thereby selected plants were further reduced to five and three BC_1_F_1_ plants, respectively. The selected plants were backcrossed to their recurrent parent and produced 465 BC_2_F_1_ seeds in Pusa44 and 424 BC_2_F_1_ seeds in Sarjoo52. The BC_2_F_1_ population was subjected to foreground and recombinant selection and six desired plants from Pusa44 and 11 desired plants from Sarjoo52 were acquired. The selected plants of BC_2_F_1_ were further backcrossed in order to increase the proportion of recurrent parental genome and produced BC_3_F_1_ seeds. A total of 725 BC_3_F_1_ seeds from Pusa44 and 850 seeds from Sarjoo52 were harvested. The BC_3_F_1_ seeds were sown and later plants were screened for desired locus and we got two desirable plants from Pusa44 cross and two desirable plants from Sarjoo52 cross. We selected plants with homozygous for recombinant markers RM493 and G11A for recurrent parent and heterozygous for target *Saltol* QTL marker RM3412 and AP3206. These plants were self-fertilized to produce BC_3_F_2_ seeds. Seeds of selected BC_3_F_2_ plants along with parents were sown in field. About 675 plants from Pusa44 and 705 plants from Sarjoo52 were screened for foreground and recombinant selection with markers viz; RM3412, AP3206 RM493 and G11A and 50 plants from Pusa44 and 62 plants from Sarjoo 52 were found desirable. Finally, 20 BC_3_F_2_ plants from PUSA 44 and 25 BC_3_F_2_ plants from Sarjoo 52 were selected. Selected plants were self-fertilized to produce BC_3_F_3_ seeds and advanced to BC_3_F_4_. At last a total of 10 desired NILs of Pusa44 and eight NILs Sarjoo52 of were found completely homozygous for *Saltol* linked marker RM3412 and AP3206 and recombinant marker RM493 and G11A (**Figures 3** and **4**). Finally 10 NILs from Pusa44 and 8 NILs from Sarjoo52 were selected based on stringent phenotyping screening (**Table 2**) and analysis of RPG recovery using 50K SNP chip. NILs were selected based on the performance in non-saline and saline stress conditions for agro-morphological traits. We selected NILs those were similar to recurrent parent for agro-morphological traits and similar to donor parent for salinity tolerance (salt injury score). Sarjoo52 NILs have recovered 94.51–98.31% and Pusa44 NILs have recovered 94.83–98.38% of parental genome, indicating high RPG recovery.

**Table 2:** Number of seed produced and desired plant through backcross selection of plants based on molecular and phenotypic screening in development of *Saltol* NILs of Pusa44 and Sajoo52.

Generation	Pusa44		Sarjoo52	
	Total number of seeds produces	No. of desired plant	Total number of seeds produces	No. of desired plant
F1	300	250	183	150
BC1F1	407	5	938	3
BC2F1	465	6	424	11
BC3F1	725	2	850	2
BC3F2	675	20	700	25
BC3F3	20	20	33	33
BC3F4	10	10	8	8

### Performance of NILs for Morphological Traits at Seedling Stage in Saline Stress

The NILs along with their respective parents were evaluated for seedling stage salinity tolerance in hydroponics and microplots (6 m × 3 m). Combined analyses of variances for developed NILs were computed by comparing the NILs × location interactions for each character and were combined for further analyses. The analysis of variance showed significant variation among developed NILs of both populations in the tested environments (**Tables 3** and **4**). Ten NILs of Pusa44 × FL478 and eight NILs of Sarjoo52 × FL478 were screened under saline conditions (E.C. ~8.0 dSm^−1^). Under salt stress condition, recurrent parent Pusa44 exhibited highly susceptible (scored 8–9) reaction, and Sarjoo52 showed susceptible (scored 7–8). On the other hand donor parent FL478 has performed well and exhibited tolerant reaction (scored 3) under salt stress conditions. Pusa44 NILs namely, PU241 and PU252 were found as tolerant lines with salt injury score of 3 and PU99, PU176, PU200, PU215, PU229, PU240, PU244, PU263 showed moderate salt tolerance with salt injury score 5 in both hydroponics and microplot condition (**Table 5**). Similarly, Sarjoo52 NILs namely, SAR39, SAR23, SAR136 and SAR17 were found as tolerant lines with salt injury score of 3 while, NILs SAR87, SAR122, SAR35 and SAR77 showed moderate salt tolerance with salt injury score of 5 (**Table 6**). The average shoot and root length in NILs of Pusa44 were skewed towards the Pusa44 parents but less than FL478. Similarly, average root and shoot length among Sarjoo52 NILs was on par with of Sarjoo52.

**Table 3:** Combined analysis of variance of different traits in *Saltol* NILs of Pusa44 under saline stress.

Source of Variation	df	Mean sum of squares				
		Salt injury score	Shoot Length	Root Length	Na^+^ Content	K^+^ Content
**Replication**	2	0.001	0.72	2.47	0.082	4.347
**NILs**	11	15.09	191.07**	33.307**	148.933**	24.008**
**Location**	1	0.001**	460.05**	468.690**	276.908**	0.055
**NILs × Location**	11	0.01	0.129	0.299	1.035	3.244
**Error**	46	0.0001	0.871	2.015	3.9376	1.7893
**Total**	71	2.33	36.68	13.183	29.688	5.504

**Significant at 0.01 level of probability.

**Table 4:** Combined analysis of variance of different traits in *Saltol* NILs of Sarjoo52 under saline stress.

Source of Variation	df	Mean sum of squares				
		Salt injury score	Shoot Length	Root Length	Na^+^ Content	K^+^ Content
**Replication**	2	0.001	229.558**	44.720**	20.951**	5.535
**NILs**	9	11.733	120.824**	15.631*	48.824**	35.634**
**Location**	1	0.001**	0.030	378.424**	360.150**	130.095**
**NILs × Location**	11	0.01	8.102	3.041	25.783*	22.348**
**Error**	38	0.001	14.021	4.516	8.944	2.358
**Total**	59	1.789	36.48	13.686	23.957	12.757

**Significant at 0.01 level of probability, *Significant at 0.05 level of probability.

**Table 5:** Performance of Pusa44 Saltol-introgressed NILs for salinity tolerance at seedling stage under hydroponics and microplot conditions. Recurrent parent genome (RPG) and phenotypic recovery of NILs based on 50K SNP analysis and DUS characterization, respectively.

NILs	Salt injury Score		Shoot Length (cm)		Root Length (cm)		Na^+^ Content (mM/g dry weight)		K^+^ Content mM/g dry weight		Phenotypic recovery (%)	Genotypic genome recovery (%)
	GH	MP	GH	MP	GH	MP	GH	MP	GH	MP		
PU-215	5	5	24.80	19.80	11.00	6.00	19.25	23.25	15.15	13.15	94.00	98.08
PU-176	5	5	25.85	20.85	12.50	7.50	27.95	30.95	15.10	16.60	86.00	97.13
PU-200	5	5	24.50	19.50	10.40	5.40	17.60	21.60	12.30	12.30	94.00	98.38
PU-240	5	5	22.25	17.25	10.00	5.00	22.20	26.20	12.95	14.95	88.00	97.88
PU-229	5	5	26.35	21.35	11.50	6.50	19.80	23.80	15.75	13.75	96.00	97.86
PU-241	3	3	27.35	22.35	16.25	11.25	22.75	26.75	15.45	15.95	84.00	94.83
PU-263	5	5	23.25	18.25	7.85	2.85	13.95	17.95	9.75	10.25	98.00	98.21
PU-99	5	5	23.15	18.15	8.25	3.25	19.05	23.05	13.10	13.10	96.00	98.10
PU-244	5	5	22.75	17.75	8.45	3.45	21.85	25.85	13.15	15.15	86.00	95.91
PU-252	3	3	23.35	18.35	10.90	5.90	19.45	23.45	13.95	13.45	96.00	96.32
Pusa44	9	9	21.75	16.75	10.75	5.75	31.30	35.30	12.80	9.30		
FL478	3	3	42.45	37.45	13.25	8.25	14.15	18.15	10.95	10.95		
Mean	4.77	4.77	25.65	20.65	10.93	5.93	20.78	24.69	13.36	13.24		
SEM	0.45	0.45	1.12	1.12	0.54	0.54	1.08	1.04	0.47	0.50		
CV%	29.65	29.65	21.87	26.38	23.95	22.63	25.29	20.13	17.99	17.77		

GH, Glass house in hydroponic solution; MP, in micro plot condition.

**Table 6:** Performance of Sarjoo52 Saltol-introgressed NILs for salinity tolerance at seedling stage under hydroponics and microplot conditions. Recurrent parent genome (RPG) and phenotypic recovery of NILs based on 50K SNP analysis and DUS characterization, respectively.

NILs	Salt Injury Score		Shoot Length (cm)		Root Length (cm)		Na^+^ Content (mM/g dry weight)		K^+^ Content (mM/g dry weight)		Phenotypic genome recovery (%)	Genotypic genome recovery (%)
	GH	MP	GH	MP	GH	MP	GH	MP	GH	MP		
SAR122	5	5	33.00	29.00	10.90	6.90	20.15	24.15	14.95	11.95	100.00	98.00
SAR77	5	5	31.50	27.50	12.50	8.50	22.05	26.05	13.05	13.05	92.00	97.15
SAR39	3	3	32.00	28.00	10.25	6.25	19.80	23.80	15.85	12.85	94.00	98.14
SAR23	3	3	28.75	24.75	10.10	6.10	23.35	27.35	17.50	14.50	98.00	94.51
SAR136	3	3	34.00	30.00	10.75	6.75	23.95	27.95	18.75	15.75	96.00	97.70
SAR87	3	3	35.60	31.60	13.50	9.50	22.10	26.10	18.80	15.80	98.00	98.31
SAR17	5	5	32.25	28.25	11.60	7.60	30.60	34.60	19.00	16.00	96.00	98.28
SAR35	5	5	32.85	28.85	12.45	8.45	17.35	21.35	14.15	11.15	98.00	97.43
Sarjoo52	7	7	31.75	27.75	11.10	7.10	21.90	25.90	11.20	8.20		
FL478	3	3	46.30	42.30	16.60	12.60	15.00	19.00	10.60	10.60		
Mean	4.26	4.26	33.80	29.80	11.98	7.98	21.63	25.63	15.39	12.99		
SEM	0.44	0.44	1.08	1.08	0.47	0.47	0.96	0.96	0.76	0.67		
CV%	33.29	33.29	14.29	15.82	17.45	25.22	19.92	16.36	22.08	21.71		

GH, Glass house in hydroponic solution; MP, in microplot condition.

### Performance of NILs for Physiological Traits at Seedling Stage in Saline Stress

Significant variation in Na^+^ and K^+^ content under salt stress condition among parents and their respective NILs was found during the physiological analysis of dried plant samples. Na^+^ content in plant samples of Pusa44 (31.30 mM/g of dry weight) was much higher than that of FL478 (14.15 mM/g of dry weight) under saline stress, which inherited the character from one of its parent Pokalli to exclude Na^+^ from its transpirational stream. On the other hand K^+^ content in FL478 was almost similar to that of Pusa44. But the Na^+^/K^+^ (1.29) ratio in FL478 was almost half to Na^+^/K^+^ ratio of Pusa44 (2.45) which could be attributed to low Na^+^ content in FL478. Among the Pusa44 NILs, the cationic (Na^+^) content was ranged from 13.95 to 27.9 mM/g of dry weight. All the Pusa44 NILs exhibited lesser Na^+^ concentration than recurrent parent and whereas, except PU263 (13.95 mM/g of dry weight) all NILs having more Na^+^ concentration than donor parent FL478. Similarly, potassium concentration of all the NILs was higher than both the parents except the lines PU263 (9.75 mM/g of dry weight) and PU200 (12.30 mM/g of dry weight). The Na^+^/K^+^ ratio was too high in Pusa44 (2.45) parent compared to salt tolerant line FL478 (1.29) and the lowest Na^+^/K^+^ ratio was observed in PU229 NILs (1.26), it is lesser than donor parent. In the cross of Sarjoo52 and FL478 again significant variation was found in cationic content of both parents. At EC ~8.0 dSm^−1^ high amount of Na^+^ content in plant samples of Sarjoo52, whereas K^+^ content was almost similar in both parents. Sarjoo52 NILs has comparatively similar Na^+^ content as that of Sarjoo52 but higher than FL478, but the K^+^ content was significantly higher in the NILs as compared to both the parents which leads to low ratio of Na^+^/K^+^. The NIL SAR17 exhibited highest Na^+^ content (30.60 mM/g of dry weight) while, NIL SAR136 exhibited highest potassium content (22.25 mM/g of dry weight). Among the Sarjoo52 NILs SAR35 and SAR77 exhibited low Na^+^ and K^+^ content, respectively. All the Sarjoo52 NILs exhibited low Na^+^/K^+^ than donor parent FL478 except SAR77 and SAR17. The SAR136 exhibited lowest Na^+^/K^+^ of 1.08. Na^+^/K^+^ ratio in high salt tolerant NILs with salt injury score of 3, ranged from 1.08 to 1.33, whereas NILs with salt injury score of 5 has Na^+^/K^+^ in range of 1.23–1.69.

### Recovery of NILs With Respect to Recurrent Parents

**Figure 5:**
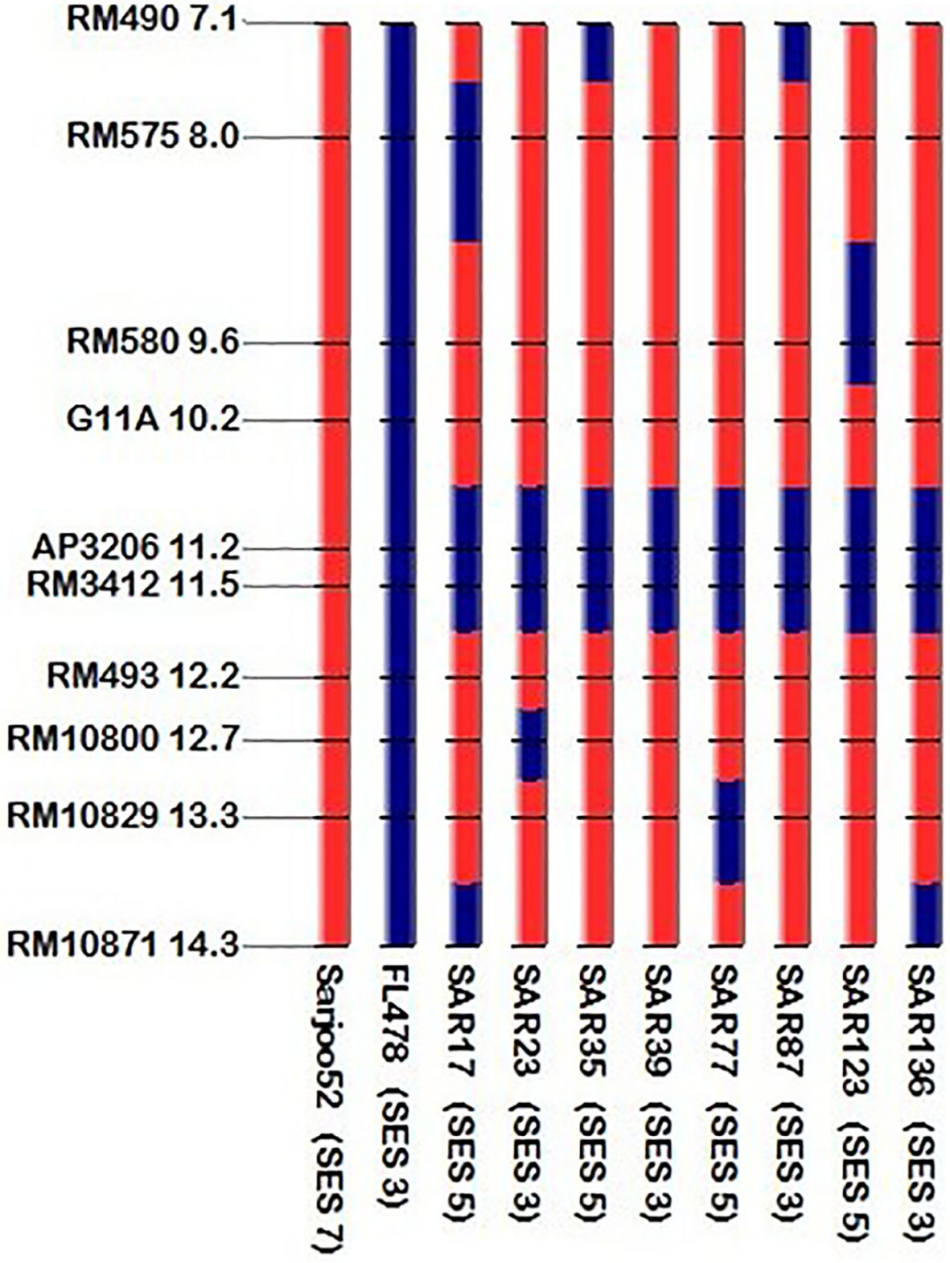
Haplotypic representation at target locus of *Saltol* introgressed BC3F4 line of Sarjoo52 along with parents.

**Figure 6:**
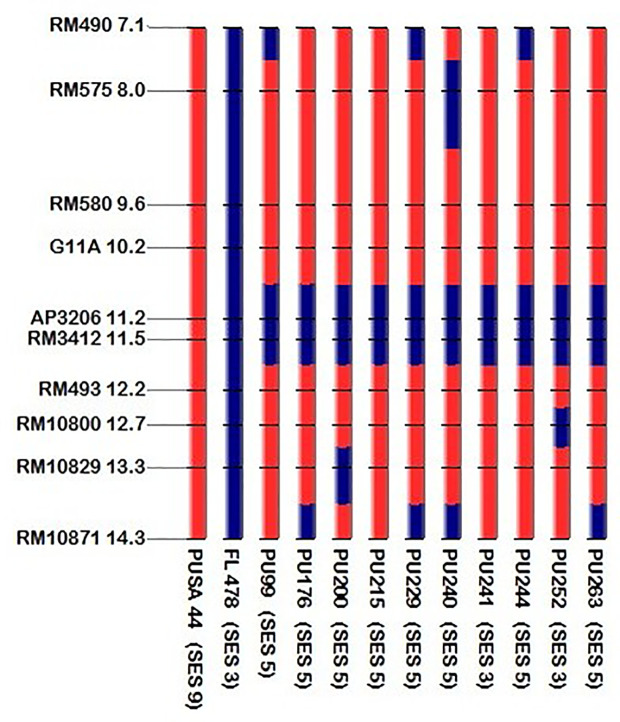
Haplotypic representation at target locus of *Saltol* introgressed BC3F4 line of Pusa44 along with parents.

Based on the field evaluation of NILs of both populations (Sarjoo52 × FL478) and (Pusa44 × FL478) it was found that the developed NILs were highly similar to their recurrent parents. On the basis of 50 agro-morphological DUS parameters, the percent recovery in NILs of Sarjoo52 × FL478 population ranged from 92 to 100% and in Pusa44 × FL478 population the percent recovery ranged from 84 to 98% when compared with respective recurrent parents (**Supplementary Table 1**). The target locus of the developed NILs was compared with that of their recurrent parent using micro-satellite markers including foreground and recombinant markers to mark the precise transfer of *Saltol* QTL (**Figures 5** and **6**). The parental genome recovery percentage at molecular level was estimated in BC_3_F_4_ NILs using 50K SNP chip. Based on the SNP analysis Sarjoo52 NILs have recovered 94.51–98.31% and Pusa44 NILs have recovered 94.83–98.38% of parental genome, indicating high RPG recovery. It confirms that the developed NILs were genotypically and phenotypically similar to the recurrent parents in addition of *Saltol* QTL from donor parent.

## Discussion

Salinity has been a major concern for rice production as cultivable land is under constant threat of saline due to excess use of irrigation water coupled with poor drainage system. Salinity stress affect rice grain yield from 20 to 100% depends on the stress level and duration of rice exposed to saline stress (Krishnamurthy et al., 2015a). Fortunately, vast genetic variability in rice in response to soil salinity makes possible to develop saline tolerant rice varieties (Akbar et al., 1972; Krishnamurthy et al., 2016c; Krishnamurthy et al., 2017; Krishnamurthy et al., 2019a; Krishnamurthy et al., 2019b; Krishnamurthy et al., 2019c). Sarjoo52 and Pusa44 are highly preferred rice cultivars among farmers for their high yielding potential under normal field condition. However, increasing soil salinity has forced farmer to minimize the area of these preferred mega varieties under cultivation. Continuous efforts are being made toward developing salt tolerant varieties through conventional breeding method (Krishnamurthy et al., 2019a; Krishnamurthy et al., 2019b; Krishnamurthy et al., 2019c). However, the problem with conventional method of breeding is that the developed progenies acquired unwanted traits due to linkage drag and possible negative effects on yield and grain quality traits of rice (Gregorio et al., 2002; Ismail et al., 2007; Thomson et al., 2010). Many researchers has introgressed the *Saltol* QTL in to different rice varieties Huyen et al. (2012) in AS996, Babu et al. (2017b) in PB1121, Geetha et al. (2017) in ADT43. Therefore the present effort was made to improve these cultivars using MABB method.

The present study was successful in introgressing *Saltol* QTL in high yielding varieties (Pusa44, Sarjoo52) and simultaneously restricted any linkage drag using marker assisted breeding approach with careful foreground, recombinant selection and background selection. The halplotype of both recipient parents differs from donor parent FL478 at four markers within *Saltol* region which includes RM3412, RM493, AP3206 and G11A. RM3412 and AP3206 were recognized as tightly linked *Saltol* markers while G11A and RM493 were selected as recombinant marker to mark the precise transfer *Saltol*. The RPG recovery in NILs of both crosses was achieved by three backcrosses with recurrent parents followed by selfing of BC_3_F_1_ lines for four generations which leads to homozygosity of *Saltol* allele. Even though the developed BC_3_F_4_ lines possesses high RPG recovery percentage but still these line has residual donor genome of 1.62–5.51%. In spite this donor region, the impact on the agronomic characters of developed NILs were negligible. In plant breeding principle, three back crosses recovered the genome by 93.75%. However, use of markers helps to enhanced it upto 98.38%. Morphological and physiochemical analysis of plant samples has revealed differential response to salinity among developed NILs. Two BC_3_F_4_ lines derived from Pusa44 × FL478 viz., PU241 and PU252 (SES score 3) were found to be more tolerant than other NILs. On the other hand NILs from Sarjoo52 × FL478 cross designated SAR39, SAR23, SAR136 and SAR87 performed very well under saline condition with salt injury score of three. Even after achieving homozygosity at *Saltol* region, the plant exhibit different ionic content and differential salinity tolerance with respect to FL478. This response to salinity could be attributed to the complexity of salt tolerance character. The other reason behind this variation could be explained by the fact that *Saltol* region from donor parent might be affected by the genetic background of recipient parents.

The *Saltol* QTL comprises multiple genes associated with salt tolerance which includes membrane transporters, signal transducers, transcriptional factors. However, Ren et al. (2005) explained its role in maintaining good Na^+^/K^+^ homoeostasis and similar homoeostasis was observed in developed NILs. In Pusa44 NILs the Na^+^ content was as low as that of FL478 while K^+^ content was higher than FL478. The Pusa44 NILs decreased the Na content by 2 folds as compared to recurrent parent (Pusa44) and Sarjoo52 NILs decreased by 1.23 folds as compared to its recurrent parent (Sarjoo 52). In Sarjoo52, NILs the Na^+^ content was more inclined toward Sarjoo52 which was significantly higher than FL478. But interestingly the K^+^ content in NILs was much higher than both parents and in NIL SAR136 K^+^ content was more than double compared to FL478. However, the Na^+^/K^+^ ratio was more skewed toward FL478 which explains the salt tolerance characters in theses developed NILs. This indicated that the introgressed region in NILs have significant role in Na^+^ and K^+^ homoeostasis. Furthermore, DUS (Distinctiveness, Uniformity, Stability) characterization revealed the similarity of these NILs with their recurrent parents. The traits used to know the phenotypic differences between NILs and parents thereby find the similarity and differences. In this context we are employed backcross breeding method to improve the Pusa44 and Sarjoo52 mega varieties by marker assisted introgression of QTLs from donor parent FL478. If we employing DUS test to compare NILs and recurrent Parents, as phenotypic differences between NILs and parents is less indicated the more similarity with recurrent parents. The developed NILs will be as similar to the parental lines except for the salt tolerance.

The present study was successful in incorporation of seedling stage salinity tolerant QTLs in two high yielding varieties i.e. Pusa44 and Sarjoo52 through marker assisted breeding. The developed lines from both the recurrent parent acquired salinity tolerance at seedling stage and simultaneously these lines have achieved high recurrent parent genome recovery during field evaluation and background selection. The *Saltol* NIL's have developed good ionic balance during physiological analysis of rice samples under salt stressed conditions with fair amount of K^+^ ions in plant samples. The developed lines will be further evaluated on multiple locations under target saline environment before they are released for commercial production in farmer's field. The present study has formed a blue chart for future breeding programs aimed for development of salt tolerant varieties highly suitable for salt affected areas.

## Data Availability Statement

All datasets generated for this study are included in the article/**Supplementary Materials**.

## Author Contributions

SK, NS, and PS conceptualized and designed the experiment. SK, PP, BL, and SR conducted the experiments. AW and BL did the field evaluations. PP, AW, and BL conducted the hydroponics screening of developed NILs. SR and PP isolated the DNA and did molecular screening. NS carried out background marker screening. SK analyzed the data. The manuscript was prepared by SK, AW, and BL. Revision of the manuscript was done by SK, NS, and PS.

## Funding

The authors acknowledge the generous funding for the project “From QTL to Variety” (Grant No. BT/PR/14544/AGR/02/745/2010) through Department of Biotechnology, Government of India.

## Conflict of Interest

The authors declare that the research was conducted in the absence of any commercial or financial relationships that could be construed as a potential conflict of interest.
