# Supplementary material for: Introgressed Saltol QTL Lines Improves the Salinity Tolerance in Rice at Seedling Stage
Source: Front Plant Sci. 2020 Jun 11;11:833. doi: 10.3389/fpls.2020.00833 (PMC7300257; doi:10.3389/fpls.2020.00833)
Supplement: Supplementary file 1 [file Table_1.docx]

**Supplementary table 1 : DUS characterization of Pusa44 NILs and Sarjoo52 NILs along with their parents in 2016, 2017 and 2018.**

| **Characters** | **Pusa44** | | | **FL478** | | | **PU-215** | | | **PU-176** | | |  |  |  |  |
| --- | --- | --- | --- | --- | --- | --- | --- | --- | --- | --- | --- | --- | --- | --- | --- | --- |
|  | **2016** | **2017** | **2018** | **2016** | **2017** | **2018** | **2016** | **2017** | **2018** | **2016** | **2017** | **2018** |  |  |  |  |
| Coleoptile: color | Green | Green | Green | Green | Green | Green | Green | Green | Green | Green | Green | Green |  |  |  |  |
| Basal leaf: sheath color | Green | Green | Green | Green | Green | Green | Green | Green | Green | Green | Green | Green |  |  |  |  |
| Leaf: intensity of green color | Medium | Medium | Medium | Medium | Medium | Medium | Medium | Medium | Medium | Medium | Medium | Medium |  |  |  |  |
| Leaf: anthocyanin coloration | Present | Present | Present | Present | Present | Present | Present | Present | Present | Present | Present | Present |  |  |  |  |
| Leaf : Dist of anthocynin coloration | On tips only | On tips only | On tips only | On tips only | On tips only | On tips only | On tips only | On tips only | On tips only | On tips only | On tips only | On tips only |  |  |  |  |
| Leaf sheath: anthocyanin coloration | Absent | Absent | Absent | Absent | Absent | Absent | Absent | Absent | Absent | Absent | Absent | Absent |  |  |  |  |
| Leaf: pubescence of blade surface | Weak | Weak | Weak | Weak | Weak | Weak | Weak | Weak | Weak | Weak | Weak | Weak |  |  |  |  |
| Leaf auricles | Present | Present | Present | Present | Present | Present | Present | Present | Present | Present | Present | Present |  |  |  |  |
| Anthocynin coloration of auricles | Colorless | Colorless | Colorless | Colorless | Colorless | Colorless | Colorless | Colorless | Colorless | Colorless | Colorless | Colorless |  |  |  |  |
| Leaf: collar | Present | Present | Present | Present | Present | Present | Present | Present | Present | Present | Present | Present |  |  |  |  |
| Leaf: antho coloration of collar | Absent | Absent | Absent | Absent | Absent | Absent | Absent | Absent | Absent | Absent | Absent | Absent |  |  |  |  |
| Leaf ligule | Present | Present | Present | Present | Present | Present | Present | Present | Present | Present | Present | Present |  |  |  |  |
| Shape of ligule | Split | Split | Split | Split | Split | Split | Split | Split | Split | Split | Split | Split |  |  |  |  |
| Leaf: color of ligule | White | White | White | White | White | White | White | White | White | White | White | White |  |  |  |  |
| Leaf: length of blade | Medium | Medium | Medium | #### | #### | #### | Medium | Medium | Medium | Medium | Medium | Medium |  |  |  |  |
| Leaf: width of blade | Narrow | Narrow | Narrow | Medium | Medium | Medium | Medium | Medium | Medium | Narrow | Narrow | Narrow |  |  |  |  |
| Culm attitude | Erect | Erect | Erect | Semi-erect | Semi-erect | Semi-erect | Erect | Erect | Erect | Erect | Erect | Erect |  |  |  |  |
| Time of heading (50% of plants with panicles) | Medium (91-110 days) | Medium (91-110 days) | Medium (91-110 days) | Early (71-90 days) | Early (71-90 days) | Early (71-90 days) | Medium (91-110 days) | Medium (91-110 days) | Medium (91-110 days) | Early (71-90 days) | Early (71-90 days) | Early (71-90 days) |  |  |  |  |
| Spikelet: density of pubescence of lemma | Weak | Weak | Weak | Medium | Medium | Medium | Weak | Weak | Weak | Medium | Medium | Medium |  |  |  |  |
| Male sterlity | Absent | Absent | Absent | Absent | Absent | Absent | Absent | Absent | Absent | Absent | Absent | Absent |  |  |  |  |
| Lemma: anth coloration of keel | Absent | Absent | Absent | Absent | Absent | Absent | Absent | Absent | Absent | Absent | Absent | Absent |  |  |  |  |
| Lemma: anthocyanin coloration of area below apex | Absent | Absent | Absent | Absent | Absent | Absent | Absent | Absent | Absent | Absent | Absent | Absent |  |  |  |  |
| Lemma: anthocyanin coloration of apex | Absent | Absent | Absent | Absent | Absent | Absent | Absent | Absent | Absent | Absent | Absent | Absent |  |  |  |  |
| Spikelet: color of stigma | White | White | White | White | White | White | White | White | White | White | White | White |  |  |  |  |
| Stem: thickness | Medium | Medium | Medium | Thick | Thick | Thick | Medium | Medium | Medium | Medium | Medium | Medium |  |  |  |  |
| Stem: length (in cm) | Very short (<91 ) | Very short (<91 ) | Very short (<91 ) | Short (91-110 ) | Short (91-110 ) | Short (91-110 ) | Very short (<91 ) | Very short (<91 ) | Very short (<91 ) | Very short (<91 ) | Very short (<91 ) | Very short (<91 ) |  |  |  |  |
| Stem: anthocyanin coloration of nodes | Absent | Absent | Absent | Absent | Absent | Absent | Absent | Absent | Absent | Absent | Absent | Absent |  |  |  |  |
| Stem: anthocynin coloration of internodes | Absent | Absent | Absent | Absent | Absent | Absent | Absent | Absent | Absent | Absent | Absent | Absent |  |  |  |  |
| Panicle: length of main axis (in cm) | Long (26-30 ) | Long (26-30 ) | Long (26-30 ) | Long (26-30 ) | Long (26-30 ) | Long (26-30 ) | Long (26-30 ) | Long (26-30 ) | Long (26-30 ) | Medium (21-25) | Medium (21-25) | Medium (21-25) |  |  |  |  |
| Flag leaf: attitude of blade (late observation) | Straight | Straight | Straight | Straight | Straight | Straight | Straight | Straight | Straight | Straight | Straight | Straight |  |  |  |  |
| Panicle: curvature of main axis | Semi-straight | Semi-straight | Semi-straight | Drooping-deflexed | Drooping-deflexed | Drooping-deflexed | Semi-straight | Semi-straight | Semi-straight | Drooping-deflexed | Drooping-deflexed | Drooping-deflexed |  |  |  |  |
| Panicle: number per plant | Few (<11) | Few (<11) | Few (<11) | Few (<11) | Few (<11) | Few (<11) | Medium (11-20) | Medium (11-20) | Medium (11-20) | Few (<11) | Few (<11) | Few (<11) |  |  |  |  |
| Spikelet : color of tip of lemma | Yellowish | Yellowish | Yellowish | Yellowish | Yellowish | Yellowish | Yellowish | Yellowish | Yellowish | Yellowish | Yellowish | Yellowish |  |  |  |  |
| Lemma and palea color | Straw | Straw | Straw | Straw | Straw | Straw | Straw | Straw | Straw | Straw | Straw | Straw |  |  |  |  |
| Panicle : awns | Absent | Absent | Absent | Present | Present | Present | Absent | Absent | Absent | Absent | Absent | Absent |  |  |  |  |
| Panicle: presence of secondary branching | Present | Present | Present | Present | Present | Present | Present | Present | Present | Present | Present | Present |  |  |  |  |
| Panicle: secondary branching | Weak | Weak | Weak | Strong | Strong | Strong | Weak | Weak | Weak | Strong | Strong | Strong |  |  |  |  |
| Panicle: attitude of branches | Semi-erect | Semi-erect | Semi-erect | Semi-erect | Semi-erect | Semi-erect | Semi-erect | Semi-erect | Semi-erect | Semi-erect | Semi-erect | Semi-erect |  |  |  |  |
| Panicle: exertion | Well exerted | Well exerted | Well exerted | Well exerted | Well exerted | Well exerted | Well exerted | Well exerted | Well exerted | Well exerted | Well exerted | Well exerted |  |  |  |  |
| Time of maturity | Medium | Medium | Medium | Medium | Medium | Medium | Medium | Medium | Medium | Medium | Medium | Medium |  |  |  |  |
| Leaf: senescence | Late | Late | Late | Late | Late | Late | Late | Late | Late | Late | Late | Late |  |  |  |  |
| Sterile lemma: color | Straw | Straw | Straw | White | White | White | Straw | Straw | Straw | White | White | White |  |  |  |  |
| Grain: Weight of 1000 fully developed grains | Low (< 15-20g) | Low (< 15-20g) | Low (< 15-20g) | Low (< 15-20g) | Low (< 15-20g) | Low (< 15-20g) | Low (< 15-20g) | Low (< 15-20g) | Low (< 15-20g) | Low (< 15-20g) | Low (< 15-20g) | Low (< 15-20g) |  |  |  |  |
| Grain: length | Medium (8.6-10.5mm) | Medium (8.6-10.5mm) | Medium (8.6-10.5mm) | Medium (8.6-10.5mm) | Medium (8.6-10.5mm) | Medium (8.6-10.5mm) | Medium (8.6-10.5mm) | Medium (8.6-10.5mm) | Medium (8.6-10.5mm) | Medium (8.6-10.5mm) | Medium (8.6-10.5mm) | Medium (8.6-10.5mm) |  |  |  |  |
| Grain: width | Medium  (2.6-3.0mm) | Medium  (2.6-3.0mm) | Medium  (2.6-3.0mm) | Medium  (2.6-3.0mm) | Medium  (2.6-3.0mm) | Medium  (2.6-3.0mm) | Narrow(2.1-2.5mm) | Narrow(2.1-2.5mm) | Narrow(2.1-2.5mm) | Narrow(2.1-2.5mm) | Narrow(2.1-2.5mm) | Narrow(2.1-2.5mm) |  |  |  |  |
| Decorticated grain: length | Short (6.1-8.5mm) | Short (6.1-8.5mm) | Short (6.1-8.5mm) | Short (6.1-8.5mm) | Short (6.1-8.5mm) |  |  |  |  |  |  | Short (6.1-8.5mm) |  |  |  |  |
| Decorticated grain: width | Medium -2.0-2.5 | Medium -2.0-2.5 | Medium -2.0-2.5 | Medium -2.0-2.11 | Medium -2.0-2.11 | Medium -2.0-2.11 | Medium -2.0-2.26 | Medium -2.0-2.26 | Medium -2.0-2.26 | Medium -2.0-2.6 | Short (6.1-8.5mm) | Short (6.1-8.5mm) | Short (6.1-8.5mm) | Short (6.1-8.5mm) | Short (6.1-8.5mm) | Short (6.1-8.5mm) |
| Decorticated grain: shape (in lateral view) | Medium -slender | Medium -slender | Medium -slender | Medium -slender | Medium -slender | Medium -slender | Medium -slender | Medium -slender | Medium -slender | Medium -slender | Medium -slender | Medium -slender |  |  |  |  |
| Decorticated grain: color | White | White | White | Brown | Brown | Brown | White | White | White | White | White | White |  |  |  |  |
| Decorticated grain aroma | Absent | Absent | Absent | Absent | Absent | Absent | Absent | Absent | Absent | Absent | Absent | Absent |  |  |  |  |

| **Characters** | **PU-200** | | | **PU-240** | | | **PU-229** | | | **PU-241** | | |
| --- | --- | --- | --- | --- | --- | --- | --- | --- | --- | --- | --- | --- |
|  | **2016** | **2017** | **2018** | **2016** | **2017** | **2018** | **2016** | **2017** | **2018** | **2016** | **2017** | **2018** |
| Coleoptile: color | Green | Green | Green | Green | Green | Green | Green | Green | Green | Green | Green | Green |
| Basal leaf: sheath color | Green | Green | Green | Green | Green | Green | Green | Green | Green | Green | Green | Green |
| Leaf: intensity of green color | Medium | Medium | Medium | Medium | Medium | Medium | Medium | Medium | Medium | Medium | Medium | Medium |
| Leaf: anthocyanin coloration | Present | Present | Present | Present | Present | Present | Present | Present | Present | Present | Present | Present |
| Leaf : Dist of anthocynin coloration | On tips only | On tips only | On tips only | On tips only | On tips only | On tips only | On tips only | On tips only | On tips only | On tips only | On tips only | On tips only |
| Leaf sheath: anthocyanin coloration | Absent | Absent | Absent | Absent | Absent | Absent | Absent | Absent | Absent | Absent | Absent | Absent |
| Leaf: pubescence of blade surface | Weak | Weak | Weak | Weak | Weak | Weak | Weak | Weak | Weak | Medium | Medium | Medium |
| Leaf auricles | Present | Present | Present | Present | Present | Present | Present | Present | Present | Present | Present | Present |
| Anthocynin coloration of auricles | Colorless | Colorless | Colorless | Colorless | Colorless | Colorless | Colorless | Colorless | Colorless | Colorless | Colorless | Colorless |
| Leaf: collar | Present | Present | Present | Present | Present | Present | Present | Present | Present | Present | Present | Present |
| Leaf: antho coloration of collar | Absent | Absent | Absent | Absent | Absent | Absent | Absent | Absent | Absent | Absent | Absent | Absent |
| Leaf ligule | Present | Present | Present | Present | Present | Present | Present | Present | Present | Present | Present | Present |
| Shape of ligule | Split | Split | Split | Split | Split | Split | Split | Split | Split | Split | Split | Split |
| Leaf: color of ligule | White | White | White | White | White | White | White | White | White | White | White | White |
| Leaf: length of blade | Medium | Medium | Medium | Medium | Medium | Medium | Medium | Medium | Medium | Medium | Medium | Medium |
| Leaf: width of blade | Narrow | Narrow | Narrow | Narrow | Narrow | Narrow | Narrow | Narrow | Narrow | Narrow | Narrow | Narrow |
| Culm attitude | Erect | Erect | Erect | Erect | Erect | Erect | Erect | Erect | Erect | Erect | Erect | Erect |
| Time of heading (50% of plants with panicles) | Medium (91-110 days) | Medium (91-110 days) | Medium (91-110 days) | Early (71-90 days) | Early (71-90 days) | Early (71-90 days) | Medium (91-110 days) | Medium (91-110 days) | Medium (91-110 days) | Early (71-90 days) | Early (71-90 days) | Early (71-90 days) |
| Spikelet: density of pubescence of lemma | Weak | Weak | Weak | Medium | Medium | Medium | Weak | Weak | Weak | Medium | Medium | Medium |
| Male sterlity | Absent | Absent | Absent | Absent | Absent | Absent | Absent | Absent | Absent | Absent | Absent | Absent |
| Lemma: anth coloration of keel | Absent | Absent | Absent | Absent | Absent | Absent | Absent | Absent | Absent | Absent | Absent | Absent |
| Lemma: anthocyanin coloration of area below apex | Absent | Absent | Absent | Absent | Absent | Absent | Absent | Absent | Absent | Absent | Absent | Absent |
| Lemma: anthocyanin coloration of apex | Absent | Absent | Absent | Absent | Absent | Absent | Absent | Absent | Absent | Absent | Absent | Absent |
| Spikelet: color of stigma | White | White | White | White | White | White | White | White | White | White | White | White |
| Stem: thickness | Medium | Medium | Medium | Medium | Medium | Medium | Medium | Medium | Medium | Medium | Medium | Medium |
| Stem: length (in cm) | Very short (<91 ) | Very short (<91 ) | Very short (<91 ) | Very short (<91 ) | Very short (<91 ) | Very short (<91 ) | Very short (<91 ) | Very short (<91 ) | Very short (<91 ) | Very short (<91 ) | Very short (<91 ) | Very short (<91 ) |
| Stem: anthocyanin coloration of nodes | Absent | Absent | Absent | Absent | Absent | Absent | Absent | Absent | Absent | Absent | Absent | Absent |
| Stem: anthocynin coloration of internodes | Absent | Absent | Absent | Absent | Absent | Absent | Absent | Absent | Absent | Absent | Absent | Absent |
| Panicle: length of main axis (in cm) | Long (26-30 ) | Long (26-30 ) | Long (26-30 ) | Medium (21-25) | Medium (21-25) | Medium (21-25) | Long (26-30 ) | Long (26-30 ) | Long (26-30 ) | Medium (21-25) | Medium (21-25) | Medium (21-25) |
| Flag leaf: attitude of blade (late observation) | Straight | Straight | Straight | Straight | Straight | Straight | Straight | Straight | Straight | Straight | Straight | Straight |
| Panicle: curvature of main axis | Semi-straight | Semi-straight | Semi-straight | Drooping-deflexed | Drooping-deflexed | Drooping-deflexed | Semi-straight | Semi-straight | Semi-straight | Drooping-deflexed | Drooping-deflexed | Drooping-deflexed |
| Panicle: number per plant | Few (<11) | Few (<11) | Few (<11) | Few (<11) | Few (<11) | Few (<11) | Medium (11-20) | Medium (11-20) | Medium (11-20) | Few (<11) | Few (<11) | Few (<11) |
| Spikelet : color of tip of lemma | Yellowish | Yellowish | Yellowish | Yellowish | Yellowish | Yellowish | Yellowish | Yellowish | Yellowish | Yellowish | Yellowish | Yellowish |
| Lemma and palea color | Straw | Straw | Straw | Straw | Straw | Straw | Straw | Straw | Straw | Straw | Straw | Straw |
| Panicle : awns | Absent | Absent | Absent | Absent | Absent | Absent | Absent | Absent | Absent | Absent | Absent | Absent |
| Panicle: presence of secondary branching | Present | Present | Present | Present | Present | Present | Present | Present | Present | Present | Present | Present |
| Panicle: secondary branching | Strong | Strong | Strong | Strong | Strong | Strong | Weak | Weak | Weak | Strong | Strong | Strong |
| Panicle: attitude of branches | Semi-erect | Semi-erect | Semi-erect | Semi-erect | Semi-erect | Semi-erect | Semi-erect | Semi-erect | Semi-erect | Semi-erect | Semi-erect | Semi-erect |
| Panicle: exertion | Well exerted | Well exerted | Well exerted | Well exerted | Well exerted | Well exerted | Well exerted | Well exerted | Well exerted | Well exerted | Well exerted | Well exerted |
| Time of maturity | Medium | Medium | Medium | Medium | Medium | Medium | Medium | Medium | Medium | Medium | Medium | Medium |
| Leaf: senescence | Late | Late | Late | Late | Late | Late | Late | Late | Late | Late | Late | Late |
| Sterile lemma: color | White | White | White | White | White | White | Straw | Straw | Straw | White | White | White |
| Grain: Weight of 1000 fully developed grains | Low (< 15-20g) | Low (< 15-20g) | Low (< 15-20g) | Low (< 15-20g) | Low (< 15-20g) | Low (< 15-20g) | Low (< 15-20g) | Low (< 15-20g) | Low (< 15-20g) | Low (< 15-20g) | Low (< 15-20g) | Low (< 15-20g) |
| Grain: length | Medium (8.6-10.5mm) | Medium (8.6-10.5mm) | Medium (8.6-10.5mm) | Medium (8.6-10.5mm) | Medium (8.6-10.5mm) | Medium (8.6-10.5mm) | Medium (8.6-10.5mm) | Medium (8.6-10.5mm) | Medium (8.6-10.5mm) | Medium (8.6-10.5mm) | Medium (8.6-10.5mm) | Medium (8.6-10.5mm) |
| Grain: width | Narrow(2.1-2.5mm) | Narrow(2.1-2.5mm) | Narrow(2.1-2.5mm) | Medium  (2.6-3.0mm) | Medium  (2.6-3.0mm) | Medium  (2.6-3.0mm) | Narrow(2.1-2.5mm) | Narrow(2.1-2.5mm) | Narrow(2.1-2.5mm) | Narrow(2.1-2.5mm) | Narrow(2.1-2.5mm) | Narrow(2.1-2.5mm) |
| Decorticated grain: length | Short (6.1-8.5mm) | Short (6.1-8.5mm) | Short (6.1-8.5mm) | Short (6.1-8.5mm) | Short (6.1-8.5mm) | Short (6.1-8.5mm) | Short (6.1-8.5mm) | Short (6.1-8.5mm) | Short (6.1-8.5mm) | Short (6.1-8.5mm) | Short (6.1-8.5mm) | Short (6.1-8.5mm) |
| Decorticated grain: width | Medium -2.0-2.12 | Medium -2.0-2.12 | Medium -2.0-2.12 | Medium -2.0-2.8 | Medium -2.0-2.8 | Medium -2.0-2.8 | Medium -2.0-2.25 | Medium -2.0-2.25 | Medium -2.0-2.25 | Medium -2.0-2.10 | Medium -2.0-2.10 | Medium -2.0-2.10 |
| Decorticated grain: shape (in lateral view) | Medium -slender | Medium -slender | Medium -slender | Medium -slender | Medium -slender | Medium -slender | Medium -slender | Medium -slender | Medium -slender | Medium -slender | Medium -slender | Medium -slender |
| Decorticated grain: color | White | White | White | White | White | White | White | White | White | White | White | White |
| Decorticated grain aroma | Absent | Absent | Absent | Absent | Absent | Absent | Absent | Absent | Absent | Absent | Absent | Absent |

| **Characters** | **PU-263** | | | **PU-99** | | | **PU-244** | | | **PU-252** | | |
| --- | --- | --- | --- | --- | --- | --- | --- | --- | --- | --- | --- | --- |
|  | **2016** | **2017** | **2018** | **2016** | **2017** | **2018** | **2016** | **2017** | **2018** | **2016** | **2017** | **2018** |
| Coleoptile: color | Green | Green | Green | Green | Green | Green | Green | Green | Green | Green | Green | Green |
| Basal leaf: sheath color | Green | Green | Green | Green | Green | Green | Green | Green | Green | Green | Green | Green |
| Leaf: intensity of green color | Medium | Medium | Medium | Medium | Medium | Medium | Medium | Medium | Medium | Medium | Medium | Medium |
| Leaf: anthocyanin coloration | Present | Present | Present | Present | Present | Present | Present | Present | Present | Present | Present | Present |
| Leaf : Dist of anthocynin coloration | On tips only | On tips only | On tips only | On tips only | On tips only | On tips only | On tips only | On tips only | On tips only | On tips only | On tips only | On tips only |
| Leaf sheath: anthocyanin coloration | Absent | Absent | Absent | Absent | Absent | Absent | Absent | Absent | Absent | Absent | Absent | Absent |
| Leaf: pubescence of blade surface | Weak | Weak | Weak | Weak | Weak | Weak | Weak | Weak | Weak | Weak | Weak | Weak |
| Leaf auricles | Present | Present | Present | Present | Present | Present | Present | Present | Present | Present | Present | Present |
| Anthocynin coloration of auricles | Colorless | Colorless | Colorless | Colorless | Colorless | Colorless | Colorless | Colorless | Colorless | Colorless | Colorless | Colorless |
| Leaf: collar | Present | Present | Present | Present | Present | Present | Present | Present | Present | Present | Present | Present |
| Leaf: antho coloration of collar | Absent | Absent | Absent | Absent | Absent | Absent | Absent | Absent | Absent | Absent | Absent | Absent |
| Leaf ligule | Present | Present | Present | Present | Present | Present | Present | Present | Present | Present | Present | Present |
| Shape of ligule | Split | Split | Split | Split | Split | Split | Split | Split | Split | Split | Split | Split |
| Leaf: color of ligule | White | White | White | White | White | White | White | White | White | White | White | White |
| Leaf: length of blade | Medium | Medium | Medium | Medium | Medium | Medium | Medium | Medium | Medium | Medium | Medium | Medium |
| Leaf: width of blade | Narrow | Narrow | Narrow | Narrow | Narrow | Narrow | Narrow | Narrow | Narrow | Narrow | Narrow | Narrow |
| Culm attitude | Erect | Erect | Erect | Erect | Erect | Erect | Erect | Erect | Erect | Erect | Erect | Erect |
| Time of heading (50% of plants with panicles) | Medium (91-110 days) | Medium (91-110 days) | Medium (91-110 days) | Medium (91-110 days) | Medium (91-110 days) | Medium (91-110 days) | Early (71-90 days) | Early (71-90 days) | Early (71-90 days) | Medium (91-110 days) | Medium (91-110 days) | Medium (91-110 days) |
| Spikelet: density of pubescence of lemma | Weak | Weak | Weak | Weak | Weak | Weak | Medium | Medium | Medium | Weak | Weak | Weak |
| Male sterlity | Absent | Absent | Absent | Absent | Absent | Absent | Absent | Absent | Absent | Absent | Absent | Absent |
| Lemma: anth coloration of keel | Absent | Absent | Absent | Absent | Absent | Absent | Absent | Absent | Absent | Absent | Absent | Absent |
| Lemma: anthocyanin coloration of area below apex | Absent | Absent | Absent | Absent | Absent | Absent | Absent | Absent | Absent | Absent | Absent | Absent |
| Lemma: anthocyanin coloration of apex | Absent | Absent | Absent | Absent | Absent | Absent | Absent | Absent | Absent | Absent | Absent | Absent |
| Spikelet: color of stigma | White | White | White | White | White | White | White | White | White | White | White | White |
| Stem: thickness | Medium | Medium | Medium | Medium | Medium | Medium | Medium | Medium | Medium | Medium | Medium | Medium |
| Stem: length (in cm) | Short (91-110 ) | Short (91-110 ) | Short (91-110 ) | Very short (<91 ) | Very short (<91 ) | Very short (<91 ) | Very short (<91 ) | Very short (<91 ) | Very short (<91 ) | Short (91-110 ) | Short (91-110 ) | Short (91-110 ) |
| Stem: anthocyanin coloration of nodes | Absent | Absent | Absent | Absent | Absent | Absent | Absent | Absent | Absent | Absent | Absent | Absent |
| Stem: anthocynin coloration of internodes | Absent | Absent | Absent | Absent | Absent | Absent | Absent | Absent | Absent | Absent | Absent | Absent |
| Panicle: length of main axis (in cm) | Long (26-30 ) | Long (26-30 ) | Long (26-30 ) | Long (26-30 ) | Long (26-30 ) | Long (26-30 ) | Medium (21-25) | Medium (21-25) | Medium (21-25) | Long (26-30 ) | Long (26-30 ) | Long (26-30 ) |
| Flag leaf: attitude of blade (late observation) | Straight | Straight | Straight | Straight | Straight | Straight | Straight | Straight | Straight | Straight | Straight | Straight |
| Panicle: curvature of main axis | Semi-straight | Semi-straight | Semi-straight | Semi-straight | Semi-straight | Semi-straight | Drooping-deflexed | Drooping-deflexed | Drooping-deflexed | Semi-straight | Semi-straight | Semi-straight |
| Panicle: number per plant | Few (<11) | Few (<11) | Few (<11) | Few (<11) | Few (<11) | Few (<11) | Few (<11) | Few (<11) | Few (<11) | Few (<11) | Few (<11) | Few (<11) |
| Spikelet : color of tip of lemma | Yellowish | Yellowish | Yellowish | Yellowish | Yellowish | Yellowish | Yellowish | Yellowish | Yellowish | Yellowish | Yellowish | Yellowish |
| Lemma and palea color | Straw | Straw | Straw | Straw | Straw | Straw | Straw | Straw | Straw | Straw | Straw | Straw |
| Panicle : awns | Absent | Absent | Absent | Absent | Absent | Absent | Absent | Absent | Absent | Absent | Absent | Absent |
| Panicle: presence of secondary branching | Present | Present | Present | Present | Present | Present | Present | Present | Present | Present | Present | Present |
| Panicle: secondary branching | Weak | Weak | Weak | Weak | Weak | Weak | Strong | Strong | Strong | Weak | Weak | Weak |
| Panicle: attitude of branches | Semi-erect | Semi-erect | Semi-erect | Semi-erect | Semi-erect | Semi-erect | Semi-erect | Semi-erect | Semi-erect | Semi-erect | Semi-erect | Semi-erect |
| Panicle: exertion | Well exerted | Well exerted | Well exerted | Well exerted | Well exerted | Well exerted | Well exerted | Well exerted | Well exerted | Well exerted | Well exerted | Well exerted |
| Time of maturity | Medium | Medium | Medium | Medium | Medium | Medium | Medium | Medium | Medium | Medium | Medium | Medium |
| Leaf: senescence | Late | Late | Late | Late | Late | Late | Late | Late | Late | Late | Late | Late |
| Sterile lemma: color | Straw | Straw | Straw | Straw | Straw | Straw | White | White | White | Straw | Straw | Straw |
| Grain: Weight of 1000 fully developed grains | Low (< 15-20g) | Low (< 15-20g) | Low (< 15-20g) | Low (< 15-20g) | Low (< 15-20g) | Low (< 15-20g) | Low (< 15-20g) | Low (< 15-20g) | Low (< 15-20g) | Low (< 15-20g) | Low (< 15-20g) | Low (< 15-20g) |
| Grain: length | Medium (8.6-10.5mm) | Medium (8.6-10.5mm) | Medium (8.6-10.5mm) | Medium (8.6-10.5mm) | Medium (8.6-10.5mm) | Medium (8.6-10.5mm) | Medium (8.6-10.5mm) | Medium (8.6-10.5mm) | Medium (8.6-10.5mm) | Medium (8.6-10.5mm) | Medium (8.6-10.5mm) | Medium (8.6-10.5mm) |
| Grain: width | Narrow(2.1-2.5mm) | Narrow(2.1-2.5mm) | Narrow(2.1-2.5mm) | Narrow(2.1-2.5mm) | Narrow(2.1-2.5mm) | Narrow(2.1-2.5mm) | Narrow(2.1-2.5mm) | Narrow(2.1-2.5mm) | Narrow(2.1-2.5mm) | Narrow(2.1-2.5mm) | Narrow(2.1-2.5mm) | Narrow(2.1-2.5mm) |
| Decorticated grain: length | Short (6.1-8.5mm) | Short (6.1-8.5mm) | Short (6.1-8.5mm) | Short (6.1-8.5mm) | Short (6.1-8.5mm) | Short (6.1-8.5mm) | Short (6.1-8.5mm) | Short (6.1-8.5mm) | Short (6.1-8.5mm) | Short (6.1-8.5mm) | Short (6.1-8.5mm) | Short (6.1-8.5mm) |
| Decorticated grain: width | Medium -2.0-2.23 | Medium -2.0-2.23 | Medium -2.0-2.23 | Medium -2.0-2.24 | Medium -2.0-2.24 | Medium -2.0-2.24 | Medium -2.0-2.7 | Medium -2.0-2.7 | Medium -2.0-2.7 | Medium -2.0-2.18 | Medium -2.0-2.18 | Medium -2.0-2.18 |
| Decorticated grain: shape (in lateral view) | Medium -slender | Medium -slender | Medium -slender | Medium -slender | Medium -slender | Medium -slender | Medium -slender | Medium -slender | Medium -slender | Medium -slender | Medium -slender | Medium -slender |
| Decorticated grain: color | White | White | White | White | White | White | White | White | White | White | White | White |
| Decorticated grain aroma | Absent | Absent | Absent | Absent | Absent | Absent | Absent | Absent | Absent | Absent | Absent | Absent |

| **Characters** | **SARJOO52** | | | **FL478** | | | | **SAR122** | | | **SAR77** | | |
| --- | --- | --- | --- | --- | --- | --- | --- | --- | --- | --- | --- | --- | --- |
|  | **2016** | **2017** | **2018** | **2016** | **2017** | | **2018** | **2016** | **2017** | **2018** | **2016** | **2017** | **2018** |
| Coleoptile: color | Green | Green | Green | Green | | Green | Green | Green | Green | Green | Green | Green | Green |
| Basal leaf: sheath color | Green | Green | Green | Green | | Green | Green | Green | Green | Green | Green | Green | Green |
| Leaf: intensity of green color | Medium | Medium | Medium | Medium | | Medium | Medium | Medium | Medium | Medium | Medium | Medium | Medium |
| Leaf: anthocyanin coloration | Present | Present | Present | Present | | Present | Present | Present | Present | Present | Present | Present | Present |
| Leaf : Dist of anthocynin coloration | On tips only | On tips only | On tips only | On tips only | | On tips only | On tips only | On tips only | On tips only | On tips only | On tips only | On tips only | On tips only |
| Leaf sheath: anthocyanin coloration | Absent | Absent | Absent | Absent | | Absent | Absent | Absent | Absent | Absent | Absent | Absent | Absent |
| Leaf: pubescence of blade surface | Weak | Weak | Weak | Weak | | Weak | Weak | Weak | Weak | Weak | Medium | Medium | Medium |
| Leaf auricles | Present | Present | Present | Present | | Present | Present | Present | Present | Present | Present | Present | Present |
| Anthocynin coloration of auricles | Colorless | Colorless | Colorless | Colorless | | Colorless | Colorless | Colorless | Colorless | Colorless | Colorless | Colorless | Colorless |
| Leaf: collar | Present | Present | Present | Present | | Present | Present | Present | Present | Present | Present | Present | Present |
| Leaf: antho coloration of collar | Absent | Absent | Absent | Absent | | Absent | Absent | Absent | Absent | Absent | Absent | Absent | Absent |
| Leaf ligule | Present | Present | Present | Present | | Present | Present | Present | Present | Present | Present | Present | Present |
| Shape of ligule | Split | Split | Split | Split | | Split | Split | Split | Split | Split | Split | Split | Split |
| Leaf: color of ligule | White | White | White | White | | White | White | White | White | White | White | White | White |
| Leaf: length of blade | Medium | Medium | Medium | #### | | #### | #### | Medium | Medium | Medium | Medium | Medium | Medium |
| Leaf: width of blade | Narrow | Narrow | Narrow | Medium | | Medium | Medium | Narrow | Narrow | Narrow | Narrow | Narrow | Narrow |
| Culm attitude | Erect | Erect | Erect | Semi-erect | | Semi-erect | Semi-erect | Erect | Erect | Erect | Semi-erect | Semi-erect | Semi-erect |
| Time of heading (50% of plants with panicles) | Medium (91-110 days) | Medium (91-110 days) | Medium (91-110 days) | Early (71-90 days) | | Early (71-90 days) | Early (71-90 days) | Medium (91-110 days) | Medium (91-110 days) | Medium (91-110 days) | Medium (91-110 days) | Medium (91-110 days) | Medium (91-110 days) |
| Spikelet: density of pubescence of lemma | Weak | Weak | Weak | Medium | | Medium | Medium | Weak | Weak | Weak | Weak | Weak | Weak |
| Male sterlity | Absent | Absent | Absent | Absent | | Absent | Absent | Absent | Absent | Absent | Absent | Absent | Absent |
| Lemma: anth coloration of keel | Absent | Absent | Absent | Absent | | Absent | Absent | Absent | Absent | Absent | Absent | Absent | Absent |
| Lemma: anthocyanin coloration of area below apex | Absent | Absent | Absent | Absent | | Absent | Absent | Absent | Absent | Absent | Absent | Absent | Absent |
| Lemma: anthocyanin coloration of apex | Absent | Absent | Absent | Absent | | Absent | Absent | Absent | Absent | Absent | Absent | Absent | Absent |
| Spikelet: color of stigma | White | White | White | White | | White | White | White | White | White | White | White | White |
| Stem: thickness | Medium | Medium | Medium | Thick | | Thick | Thick | Medium | Medium | Medium | Medium | Medium | Medium |
| Stem: length (in cm) | Short (91-110 ) | Short (91-110 ) | Short (91-110 ) | Short (91-110 ) | | Short (91-110 ) | Short (91-110 ) | Short (91-110 ) | Short (91-110 ) | Short (91-110 ) | Short (91-110 ) | Short (91-110 ) | Short (91-110 ) |
| Stem: anthocyanin coloration of nodes | Absent | Absent | Absent | Absent | | Absent | Absent | Absent | Absent | Absent | Absent | Absent | Absent |
| Stem: anthocynin coloration of internodes | Absent | Absent | Absent | Absent | | Absent | Absent | Absent | Absent | Absent | Absent | Absent | Absent |
| Panicle: length of main axis (in cm) | Long (26-30 ) | Long (26-30 ) | Long (26-30 ) | Long (26-30 ) | | Long (26-30 ) | Long (26-30 ) | Long (26-30 ) | Long (26-30 ) | Long (26-30 ) | Long (26-30 ) | Long (26-30 ) | Long (26-30 ) |
| Flag leaf: attitude of blade (late observation) | Straight | Straight | Straight | Straight | | Straight | Straight | Straight | Straight | Straight | Straight | Straight | Straight |
| Panicle: curvature of main axis | Deflexed-drooping | Deflexed-drooping | Deflexed-drooping | Drooping-deflexed | | Drooping-deflexed | Drooping-deflexed | Deflexed-drooping | Deflexed-drooping | Deflexed-drooping | Semi-straight | Semi-straight | Semi-straight |
| Panicle: number per plant | Few (<11) | Few (<11) | Few (<11) | Few (<11) | | Few (<11) | Few (<11) | Few (<11) | Few (<11) | Few (<11) | Medium (11-20) | Medium (11-20) | Medium (11-20) |
| Spikelet : color of tip of lemma | Yellowish | Yellowish | Yellowish | Yellowish | | Yellowish | Yellowish | Yellowish | Yellowish | Yellowish | Yellowish | Yellowish | Yellowish |
| Lemma and palea color | Straw | Straw | Straw | Straw | | Straw | Straw | Straw | Straw | Straw | Straw | Straw | Straw |
| Panicle : awns | Absent | Absent | Absent | Present | | Present | Present | Absent | Absent | Absent | Absent | Absent | Absent |
| Panicle: presence of secondary branching | Present | Present | Present | Present | | Present | Present | Present | Present | Present | Present | Present | Present |
| Panicle: secondary branching | Weak | Weak | Weak | Strong | | Strong | Strong | Weak | Weak | Weak | Weak | Weak | Weak |
| Panicle: attitude of branches | Semi-erect | Semi-erect | Semi-erect | Semi-erect | | Semi-erect | Semi-erect | Semi-erect | Semi-erect | Semi-erect | Semi-erect | Semi-erect | Semi-erect |
| Panicle: exertion | Well exerted | Well exerted | Well exerted | Well exerted | | Well exerted | Well exerted | Well exerted | Well exerted | Well exerted | Well exerted | Well exerted | Well exerted |
| Time of maturity | Medium | Medium | Medium | Medium | | Medium | Medium | Medium | Medium | Medium | Medium | Medium | Medium |
| Leaf: senescence | Late | Late | Late | Late | | Late | Late | Late | Late | Late | Late | Late | Late |
| Sterile lemma: color | Straw | Straw | Straw | White | | White | White | Straw | Straw | Straw | Straw | Straw | Straw |
| Grain: Weight of 1000 fully developed grains | Low (< 15-20g) | Low (< 15-20g) | Low (< 15-20g) | Low (< 15-20g) | | Low (< 15-20g) | Low (< 15-20g) | Low (< 15-20g) | Low (< 15-20g) | Low (< 15-20g) | Low (< 15-20g) | Low (< 15-20g) | Low (< 15-20g) |
| Grain: length | Short(6.1-8.5 mm) | Short(6.1-8.5 mm) | Short(6.1-8.5 mm) | Medium (8.6-10.5mm) | | Medium (8.6-10.5mm) | Medium (8.6-10.5mm) | Short(6.1-8.5 mm) | Short(6.1-8.5 mm) | Short(6.1-8.5 mm) | Short(6.1-8.5 mm) | Short(6.1-8.5 mm) | Short(6.1-8.5 mm) |
| Grain: width | Medium  (2.6-3.0mm) | Medium  (2.6-3.0mm) | Medium  (2.6-3.0mm) | Medium  (2.6-3.0mm) | | Medium  (2.6-3.0mm) | Medium  (2.6-3.0mm) | Medium  (2.6-3.0mm) | Medium  (2.6-3.0mm) | Medium  (2.6-3.0mm) | Medium  (2.6-3.0mm) | Medium  (2.6-3.0mm) | Medium  (2.6-3.0mm) |
| Decorticated grain: length | Short (6.1-8.5mm) | Short (6.1-8.5mm) | Short (6.1-8.5mm) | Short (6.1-8.5mm) | | Short (6.1-8.5mm) | Short (6.1-8.5mm) | Short (6.1-8.5mm) | Short (6.1-8.5mm) | Short (6.1-8.5mm) | Short (6.1-8.5mm) | Short (6.1-8.5mm) | Short (6.1-8.5mm) |
| Decorticated grain: width | Medium -2.0-2.52 | Medium -2.0-2.52 | Medium -2.0-2.52 | Medium -2.0-2.11 | | Medium -2.0-2.11 | Medium -2.0-2.11 | Medium -2.0-2.61 | Medium -2.0-2.61 | Medium -2.0-2.61 | Medium -2.0-2.29 | Medium -2.0-2.29 | Medium -2.0-2.29 |
| Decorticated grain: shape (in lateral view) | Medium -slender | Medium -slender | Medium -slender | Medium -slender | | Medium -slender | Medium -slender | Medium -slender | Medium -slender | Medium -slender | Medium -slender | Medium -slender | Medium -slender |
| Decorticated grain: color | White | White | White | Brown | | Brown | Brown | White | White | White | White | White | White |
| Decorticated grain aroma | Absent | Absent | Absent | Absent | | Absent | Absent | Absent | Absent | Absent | Absent | Absent | Absent |

| **Characters** | **SAR39** | | | **SAR23** | | | **SAR136** | | | **SAR87** | | |
| --- | --- | --- | --- | --- | --- | --- | --- | --- | --- | --- | --- | --- |
|  | **2016** | **2017** | **2018** | **2016** | **2017** | **2018** | **2016** | **2017** | **2018** | **2016** | **2017** | **2018** |
| Coleoptile: color | Green | Green | Green | Green | Green | Green | Green | Green | Green | Green | Green | Green |
| Basal leaf: sheath color | Green | Green | Green | Green | Green | Green | Green | Green | Green | Green | Green | Green |
| Leaf: intensity of green color | Medium | Medium | Medium | Medium | Medium | Medium | Medium | Medium | Medium | Medium | Medium | Medium |
| Leaf: anthocyanin coloration | Present | Present | Present | Present | Present | Present | Present | Present | Present | Present | Present | Present |
| Leaf : Dist of anthocynin coloration | On tips only | On tips only | On tips only | On tips only | On tips only | On tips only | On tips only | On tips only | On tips only | On tips only | On tips only | On tips only |
| Leaf sheath: anthocyanin coloration | Absent | Absent | Absent | Absent | Absent | Absent | Absent | Absent | Absent | Absent | Absent | Absent |
| Leaf: pubescence of blade surface | Weak | Weak | Weak | Weak | Weak | Weak | Weak | Weak | Weak | Weak | Weak | Weak |
| Leaf auricles | Present | Present | Present | Present | Present | Present | Present | Present | Present | Present | Present | Present |
| Anthocynin coloration of auricles | Colorless | Colorless | Colorless | Colorless | Colorless | Colorless | Colorless | Colorless | Colorless | Colorless | Colorless | Colorless |
| Leaf: collar | Present | Present | Present | Present | Present | Present | Present | Present | Present | Present | Present | Present |
| Leaf: antho coloration of collar | Absent | Absent | Absent | Absent | Absent | Absent | Absent | Absent | Absent | Absent | Absent | Absent |
| Leaf ligule | Present | Present | Present | Present | Present | Present | Present | Present | Present | Present | Present | Present |
| Shape of ligule | Split | Split | Split | Split | Split | Split | Split | Split | Split | Split | Split | Split |
| Leaf: color of ligule | White | White | White | White | White | White | White | White | White | White | White | White |
| Leaf: length of blade | Medium | Medium | Medium | Medium | Medium | Medium | Medium | Medium | Medium | Medium | Medium | Medium |
| Leaf: width of blade | Medium | Medium | Medium | Narrow | Narrow | Narrow | Narrow | Narrow | Narrow | Narrow | Narrow | Narrow |
| Culm attitude | Erect | Erect | Erect | Erect | Erect | Erect | Erect | Erect | Erect | Erect | Erect | Erect |
| Time of heading (50% of plants with panicles) | Medium (91-110 days) | Medium (91-110 days) | Medium (91-110 days) | Medium (91-110 days) | Medium (91-110 days) | Medium (91-110 days) | Medium (91-110 days) | Medium (91-110 days) | Medium (91-110 days) | Medium (91-110 days) | Medium (91-110 days) | Medium (91-110 days) |
| Spikelet: density of pubescence of lemma | Weak | Weak | Weak | Weak | Weak | Weak | Weak | Weak | Weak | Weak | Weak | Weak |
| Male sterlity | Absent | Absent | Absent | Absent | Absent | Absent | Absent | Absent | Absent | Absent | Absent | Absent |
| Lemma: anth coloration of keel | Absent | Absent | Absent | Absent | Absent | Absent | Absent | Absent | Absent | Absent | Absent | Absent |
| Lemma: anthocyanin coloration of area below apex | Absent | Absent | Absent | Absent | Absent | Absent | Absent | Absent | Absent | Absent | Absent | Absent |
| Lemma: anthocyanin coloration of apex | Absent | Absent | Absent | Absent | Absent | Absent | Absent | Absent | Absent | Absent | Absent | Absent |
| Spikelet: color of stigma | White | White | White | White | White | White | White | White | White | White | White | White |
| Stem: thickness | Medium | Medium | Medium | Medium | Medium | Medium | Medium | Medium | Medium | Medium | Medium | Medium |
| Stem: length (in cm) | Short (91-110 ) | Short (91-110 ) | Short (91-110 ) | Short (91-110 ) | Short (91-110 ) | Short (91-110 ) | Medium (111-130) | Medium (111-130) | Medium (111-130) | Medium (111-130) | Medium (111-130) | Medium (111-130) |
| Stem: anthocyanin coloration of nodes | Absent | Absent | Absent | Absent | Absent | Absent | Absent | Absent | Absent | Absent | Absent | Absent |
| Stem: anthocynin coloration of internodes | Absent | Absent | Absent | Absent | Absent | Absent | Absent | Absent | Absent | Absent | Absent | Absent |
| Panicle: length of main axis (in cm) | Long (26-30 ) | Long (26-30 ) | Long (26-30 ) | Long (26-30 ) | Long (26-30 ) | Long (26-30 ) | Long (26-30 ) | Long (26-30 ) | Long (26-30 ) | Long (26-30 ) | Long (26-30 ) | Long (26-30 ) |
| Flag leaf: attitude of blade (late observation) | Straight | Straight | Straight | Straight | Straight | Straight | Straight | Straight | Straight | Straight | Straight | Straight |
| Panicle: curvature of main axis | Semi-straight | Semi-straight | Semi-straight | Drooping-deflexed | Drooping-deflexed | Drooping-deflexed | Deflexed-drooping | Deflexed-drooping | Deflexed-drooping | Deflexed-drooping | Deflexed-drooping | Deflexed-drooping |
| Panicle: number per plant | Medium (11-20) | Medium (11-20) | Medium (11-20) | Few (<11) | Few (<11) | Few (<11) | Medium (11-20) | Medium (11-20) | Medium (11-20) | Medium (11-20) | Medium (11-20) | Medium (11-20) |
| Spikelet : color of tip of lemma | Yellowish | Yellowish | Yellowish | Yellowish | Yellowish | Yellowish | Yellowish | Yellowish | Yellowish | Yellowish | Yellowish | Yellowish |
| Lemma and palea color | Straw | Straw | Straw | Straw | Straw | Straw | Straw | Straw | Straw | Straw | Straw | Straw |
| Panicle : awns | Absent | Absent | Absent | Absent | Absent | Absent | Absent | Absent | Absent | Absent | Absent | Absent |
| Panicle: presence of secondary branching | Present | Present | Present | Present | Present | Present | Present | Present | Present | Present | Present | Present |
| Panicle: secondary branching | Weak | Weak | Weak | Weak | Weak | Weak | Weak | Weak | Weak | Weak | Weak | Weak |
| Panicle: attitude of branches | Semi-erect | Semi-erect | Semi-erect | Semi-erect | Semi-erect | Semi-erect | Semi-erect | Semi-erect | Semi-erect | Semi-erect | Semi-erect | Semi-erect |
| Panicle: exertion | Well exerted | Well exerted | Well exerted | Well exerted | Well exerted | Well exerted | Well exerted | Well exerted | Well exerted | Well exerted | Well exerted | Well exerted |
| Time of maturity | Medium | Medium | Medium | Medium | Medium | Medium | Medium | Medium | Medium | Medium | Medium | Medium |
| Leaf: senescence | Late | Late | Late | Late | Late | Late | Late | Late | Late | Late | Late | Late |
| Sterile lemma: color | Straw | Straw | Straw | Straw | Straw | Straw | Straw | Straw | Straw | Straw | Straw | Straw |
| Grain: Weight of 1000 fully developed grains | Low (< 15-20g) | Low (< 15-20g) | Low (< 15-20g) | Low (< 15-20g) | Low (< 15-20g) | Low (< 15-20g) | Low (< 15-20g) | Low (< 15-20g) | Low (< 15-20g) | Low (< 15-20g) | Low (< 15-20g) | Low (< 15-20g) |
| Grain: length | Short(6.1-8.5 mm) | Short(6.1-8.5 mm) | Short(6.1-8.5 mm) | Short(6.1-8.5 mm) | Short(6.1-8.5 mm) | Short(6.1-8.5 mm) | Short(6.1-8.5 mm) | Short(6.1-8.5 mm) | Short(6.1-8.5 mm) | Short(6.1-8.5 mm) | Short(6.1-8.5 mm) | Short(6.1-8.5 mm) |
| Grain: width | Medium  (2.6-3.0mm) | Medium  (2.6-3.0mm) | Medium  (2.6-3.0mm) | Medium  (2.6-3.0mm) | Medium  (2.6-3.0mm) | Medium  (2.6-3.0mm) | Medium  (2.6-3.0mm) | Medium  (2.6-3.0mm) | Medium  (2.6-3.0mm) | Medium  (2.6-3.0mm) | Medium  (2.6-3.0mm) | Medium  (2.6-3.0mm) |
| Decorticated grain: length | Short (6.1-8.5mm) | Short (6.1-8.5mm) | Short (6.1-8.5mm) | Short (6.1-8.5mm) | Short (6.1-8.5mm) | Short (6.1-8.5mm) | Short (6.1-8.5mm) | Short (6.1-8.5mm) | Short (6.1-8.5mm) | Short (6.1-8.5mm) | Short (6.1-8.5mm) | Short (6.1-8.5mm) |
| Decorticated grain: width | Medium -2.0-2.32 | Medium -2.0-2.32 | Medium -2.0-2.32 | Medium -2.0-2.34 | Medium -2.0-2.34 | Medium -2.0-2.34 | Medium -2.0-2.60 | Medium -2.0-2.60 | Medium -2.0-2.60 | Medium -2.0-2.53 | Medium -2.0-2.53 | Medium -2.0-2.53 |
| Decorticated grain: shape (in lateral view) | Medium -slender | Medium -slender | Medium -slender | Medium -slender | Medium -slender | Medium -slender | Medium -slender | Medium -slender | Medium -slender | Medium -slender | Medium -slender | Medium -slender |
| Decorticated grain: color | White | White | White | White | White | White | White | White | White | White | White | White |
| Decorticated grain aroma | Absent | Absent | Absent | Absent | Absent | Absent | Absent | Absent | Absent | Absent | Absent | Absent |

| **Characters** | **SAR17** | | | **SAR35** | | |
| --- | --- | --- | --- | --- | --- | --- |
|  | **2016** | **2017** | **2018** | **2016** | **2017** | **2018** |
| Coleoptile: color | Green | Green | Green | Green | Green | Green |
| Basal leaf: sheath color | Green | Green | Green | Green | Green | Green |
| Leaf: intensity of green color | Medium | Medium | Medium | Medium | Medium | Medium |
| Leaf: anthocyanin coloration | Present | Present | Present | Present | Present | Present |
| Leaf : Dist of anthocynin coloration | On tips only | On tips only | On tips only | On tips only | On tips only | On tips only |
| Leaf sheath: anthocyanin coloration | Absent | Absent | Absent | Absent | Absent | Absent |
| Leaf: pubescence of blade surface | Weak | Weak | Weak | Weak | Weak | Weak |
| Leaf auricles | Present | Present | Present | Present | Present | Present |
| Anthocynin coloration of auricles | Colorless | Colorless | Colorless | Colorless | Colorless | Colorless |
| Leaf: collar | Present | Present | Present | Present | Present | Present |
| Leaf: antho coloration of collar | Absent | Absent | Absent | Absent | Absent | Absent |
| Leaf ligule | Present | Present | Present | Present | Present | Present |
| Shape of ligule | Split | Split | Split | Split | Split | Split |
| Leaf: color of ligule | White | White | White | White | White | White |
| Leaf: length of blade | Medium | Medium | Medium | Medium | Medium | Medium |
| Leaf: width of blade | Narrow | Narrow | Narrow | Narrow | Narrow | Narrow |
| Culm attitude | Erect | Erect | Erect | Erect | Erect | Erect |
| Time of heading (50% of plants with panicles) | Medium (91-110 days) | Medium (91-110 days) | Medium (91-110 days) | Medium (91-110 days) | Medium (91-110 days) | Medium (91-110 days) |
| Spikelet: density of pubescence of lemma | Weak | Weak | Weak | Weak | Weak | Weak |
| Male sterlity | Absent | Absent | Absent | Absent | Absent | Absent |
| Lemma: anth coloration of keel | Absent | Absent | Absent | Absent | Absent | Absent |
| Lemma: anthocyanin coloration of area below apex | Absent | Absent | Absent | Absent | Absent | Absent |
| Lemma: anthocyanin coloration of apex | Absent | Absent | Absent | Absent | Absent | Absent |
| Spikelet: color of stigma | White | White | White | White | White | White |
| Stem: thickness | Medium | Medium | Medium | Medium | Medium | Medium |
| Stem: length (in cm) | Short (91-110 ) | Short (91-110 ) | Short (91-110 ) | Short (91-110 ) | Short (91-110 ) | Short (91-110 ) |
| Stem: anthocyanin coloration of nodes | Absent | Absent | Absent | Absent | Absent | Absent |
| Stem: anthocynin coloration of internodes | Absent | Absent | Absent | Absent | Absent | Absent |
| Panicle: length of main axis (in cm) | Long (26-30 ) | Long (26-30 ) | Long (26-30 ) | Long (26-30 ) | Long (26-30 ) | Long (26-30 ) |
| Flag leaf: attitude of blade (late observation) | Straight | Straight | Straight | Straight | Straight | Straight |
| Panicle: curvature of main axis | Drooping-deflexed | Drooping-deflexed | Drooping-deflexed | Deflexed-drooping | Deflexed-drooping | Deflexed-drooping |
| Panicle: number per plant | Medium (11-20) | Medium (11-20) | Medium (11-20) | Medium (11-20) | Medium (11-20) | Medium (11-20) |
| Spikelet : color of tip of lemma | Yellowish | Yellowish | Yellowish | Yellowish | Yellowish | Yellowish |
| Lemma and palea color | Straw | Straw | Straw | Straw | Straw | Straw |
| Panicle : awns | Absent | Absent | Absent | Absent | Absent | Absent |
| Panicle: presence of secondary branching | Present | Present | Present | Present | Present | Present |
| Panicle: secondary branching | Weak | Weak | Weak | Weak | Weak | Weak |
| Panicle: attitude of branches | Semi-erect | Semi-erect | Semi-erect | Semi-erect | Semi-erect | Semi-erect |
| Panicle: exertion | Well exerted | Well exerted | Well exerted | Well exerted | Well exerted | Well exerted |
| Time of maturity | Medium | Medium | Medium | Medium | Medium | Medium |
| Leaf: senescence | Late | Late | Late | Late | Late | Late |
| Sterile lemma: color | Straw | Straw | Straw | Straw | Straw | Straw |
| Grain: Weight of 1000 fully developed grains | Low (< 15-20g) | Low (< 15-20g) | Low (< 15-20g) | Low (< 15-20g) | Low (< 15-20g) | Low (< 15-20g) |
| Grain: length | Short(6.1-8.5 mm) | Short(6.1-8.5 mm) | Short(6.1-8.5 mm) | Short(6.1-8.5 mm) | Short(6.1-8.5 mm) | Short(6.1-8.5 mm) |
| Grain: width | Medium  (2.6-3.0mm) | Medium  (2.6-3.0mm) | Medium  (2.6-3.0mm) | Medium  (2.6-3.0mm) | Medium  (2.6-3.0mm) | Medium  (2.6-3.0mm) |
| Decorticated grain: length | Short (6.1-8.5mm) | Short (6.1-8.5mm) | Short (6.1-8.5mm) | Short (6.1-8.5mm) | Short (6.1-8.5mm) | Short (6.1-8.5mm) |
| Decorticated grain: width | Medium -2.0-2.36 | Medium -2.0-2.36 | Medium -2.0-2.36 | Medium -2.0-2.46 | Medium -2.0-2.46 | Medium -2.0-2.46 |
| Decorticated grain: shape (in lateral view) | Medium -slender | Medium -slender | Medium -slender | Medium -slender | Medium -slender | Medium -slender |
| Decorticated grain: color | White | White | White | White | White | White |
| Decorticated grain aroma | Absent | Absent | Absent | Absent | Absent | Absent |
